# Quantitative global lipidomics analysis of patients with ovarian cancer versus benign adnexal mass

**DOI:** 10.1038/s41598-021-97433-x

**Published:** 2021-09-13

**Authors:** Matthew F. Buas, Charles W. Drescher, Nicole Urban, Christopher I. Li, Lisa Bettcher, Nitai C. Hait, Kirsten B. Moysich, Kunle Odunsi, Daniel Raftery, Li Yan

**Affiliations:** 1 Department of Cancer Prevention and Control, Roswell Park Comprehensive Cancer Center, Elm and Carlton Streets, Buffalo, NY 14263 USA; 2 Division of Public Health Sciences, Fred Hutchinson Cancer Research Center, 1100 Fairview Ave. N, Seattle, WA 98109 USA; 3 Department of Anesthesiology and Pain Medicine, Northwest Metabolomics Research Center, University of Washington School of Medicine, 850 Republican Street, Seattle, WA 98109 USA; 4 Department of Surgical Oncology, Roswell Park Comprehensive Cancer Center, Elm and Carlton Streets, Buffalo, NY 14263 USA; 5 Department of Molecular and Cellular Biology, Roswell Park Comprehensive Cancer Center, Elm and Carlton Streets, Buffalo, NY 14263 USA; 6 Department of Gynecologic Oncology, Roswell Park Comprehensive Cancer Center, Elm and Carlton Streets, Buffalo, NY 14263 USA; 7 Department of Bioinformatics and Biostatistics, Roswell Park Comprehensive Cancer Center, Elm and Carlton Streets, Buffalo, NY 14263 USA

**Keywords:** Ovarian cancer, Tumour biomarkers, Diagnostic markers, Ovarian cancer, Cancer metabolism

## Abstract

Altered lipid metabolism has emerged as an important feature of ovarian cancer (OC), yet the translational potential of lipid metabolites to aid in diagnosis and triage remains unproven. We conducted a multi-level interrogation of lipid metabolic phenotypes in patients with adnexal masses, integrating quantitative lipidomics profiling of plasma and ascites with publicly-available tumor transcriptome data. Using Sciex Lipidyzer, we assessed concentrations of > 500 plasma lipids in two patient cohorts—(i) a pilot set of 100 women with OC (50) or benign tumor (50), and (ii) an independent set of 118 women with malignant (60) or benign (58) adnexal mass. 249 lipid species and several lipid classes were significantly reduced in cases versus controls in both cohorts (FDR < 0.05). 23 metabolites—triacylglycerols, phosphatidylcholines, cholesterol esters—were validated at Bonferroni significance (P < 9.16 × 10^–5^). Certain lipids exhibited greater alterations in early- (diacylglycerols) or late-stage (lysophospholipids) cases, and multiple lipids in plasma and ascites were positively correlated. Lipoprotein receptor gene expression differed markedly in OC versus benign tumors. Importantly, several plasma lipid species, such as DAG(16:1/18:1), improved the accuracy of CA125 in differentiating early-stage OC cases from benign controls, and conferred a 15–20% increase in specificity at 90% sensitivity in multivariate models adjusted for age and BMI. This study provides novel insight into systemic and local lipid metabolic differences between OC and benign disease, further implicating altered lipid uptake in OC biology, and advancing plasma lipid metabolites as a complementary class of circulating biomarkers for OC diagnosis and triage.

## Introduction

Ovarian cancer (OC) is a leading cause of cancer-related death among U.S. women^1^. Most OC patients are diagnosed with late-stage disease, which carries a 5-year survival rate of < 30%^1,2^. While effective screening and early detection remain long-sought translational goals, advancing our biological understanding of OC pathogenesis and identifying novel molecular readouts of the disease process represent critical intermediate objectives, with important translational implications for improved diagnosis and clinical triage of women suspected of OC, to ensure they receive needed subspecialty care^3^.

It has been known for decades that cancer cells exhibit metabolic alterations such as the Warburg effect (aerobic glycolysis), considered an adaptive response to the demands of high proliferation rates and hypoxic tumor microenvironments^4,5^. In recent years, changes in lipid metabolism have increasingly been recognized as important features of multiple cancers^6^. Lipids function as essential substrates for new cell membranes, rich fuel sources, and key signaling mediators. Increased fatty acid synthesis has historically been viewed as a major source of lipids in proliferating cancer cells^7^, and accumulating experimental evidence now supports a key role for lipid transfer and uptake pathways in OC growth and metastasis^8–11^.

The field of metabolomics studies the concentrations and fluxes of low-molecular-weight (typically < 1000 Dalton) metabolites in biofluids or tissue^12,13^. Since metabolic fluctuations lie downstream of alterations at the DNA, RNA, and protein level, metabolite profiles can provide a comprehensive and sensitive functional read-out of biological systems, by integrating information from genome, transcriptome, and proteome. In the past five years, lipidomics has emerged as a specialized sub-discipline of metabolomics, specifically focused on interrogation of lipid metabolites (the ‘lipidome’)^14,15^.

Early metabolomics studies of OC reported differences in the abundance of a wide range of metabolites, including certain lipids, in tissue and blood specimens isolated from patients with OC versus women with benign ovarian tumors or no ovarian pathology^16–22^. While intriguing, these studies were hampered by heterogeneous quantitation platforms, limited reproducibility, and minimal coverage of the lipidome. In 2015, we published the first global lipidomics analysis of OC and reported reduced levels of multiple glycerolipids and glycerophospholipids in the plasma of women with malignant as compared to benign serous ovarian tumors^23^. Though based on a single cohort of 100 patients and a relative quantitation profiling system, these results illustrated the power of lipidomics to (i) provide novel insight into systemic lipid metabolic phenotypes in OC and (ii) identify circulating lipid species with potential to boost diagnostic accuracy of existing protein markers for OC such as CA125. Subsequent independent studies using various profiling technologies have reported similar reductions in abundant lipid species in the circulation of women with OC^24–29^, but much of the focus has been on relating lipid metabolite profiles to patient prognosis and survival. Substantial gaps remain in linking blood lipidome phenotypes to distinct molecular features in cancers versus benign tumors and advancing the development of novel lipid metabolite markers to improve clinical diagnosis and triage.

In the present study, we employed a state-of-the-art quantitative lipidomics profiling platform (Sciex Lipidyzer) to conduct the most comprehensive interrogation to date of the plasma lipidome in 218 patients with malignant or benign adnexal masses, encompassing diverse tumor histologies and disease stages from two independent clinical sites. Differential gene expression analysis of ovarian tumor tissues, using public transcriptome data, provided further insight into lipid-transport-related expression signatures correlated with observed alterations in the plasma lipidome. The utility of circulating lipid metabolites to enhance the accuracy of CA125 in distinguishing between women with early-stage OC or benign adnexal mass was investigated through construction and evaluation of multivariate statistical classification models.

## Materials and methods

### Study populations

Two independent patient cohorts were included in this study to allow for validation of our findings. The first cohort (Fred Hutch: FH), analyzed in our previous study using a different profiling platform^23^, was comprised of patients who were recruited between May 2006 and August 2011 by physicians at the Swedish Cancer Institute (Swedish Medical Center, Seattle, WA) to support protocols of the Pacific Ovarian Cancer Research Consortium (POCRC), an NCI-funded Ovarian Cancer SPORE centered at the Fred Hutchinson Cancer Research Center. All included participants were post-menopausal White women who were referred for surgical excision of an adnexal mass and had no prior history of cancer. Inclusion of patients from other ethnic backgrounds was precluded by limited sample availability, reflecting reduced disease incidence. 50 cases with serous ovarian carcinoma and 50 controls with benign serous ovarian tumor were selected for analysis. Controls were frequency-matched to cases by age, body-mass index (BMI), parity, use of oral contraceptives and year of blood draw. Demographic and lifestyle characteristics were obtained through questionnaires completed by patients prior to surgery. The second cohort (Roswell Park: RP) was comprised of patients who presented to Roswell Park Comprehensive Cancer Center for evaluation and surgical excision of an adnexal mass, and were enrolled in the Roswell Park Data Bank and BioRepository (DBBR). The DBBR is a continuously operating comprehensive data and sample bank that seeks to enroll all cancer patients seen at Roswell Park, in addition to a large number of community-based cancer-free controls^30^. As part of informed consent, individuals agree to complete a standardized questionnaire, provide a blood sample, and have their data and samples linked with clinical information, when applicable. All included participants for this study were post-menopausal White women with no prior history of cancer. 60 ovarian carcinoma cases and 58 benign controls were selected for analysis, representing both serous and non-serous tumor histologies. The control group included women with benign ovarian tumors or other non-malignant conditions associated with an adnexal mass.

### Biospecimens

Blood samples from included study participants were collected prior to surgery or other treatment, under fasting (FH) or non-fasting (RP) conditions, and processed according to standard operating protocols, as described previously^23,30,31^. All blood specimens were collected into EDTA tubes and processed within four hours post-draw. Ascites specimens from a subset of 15 OC patients at Roswell Park were collected prior to surgery. Each biospecimen was assigned a unique laboratory identification number, which blinded laboratory personnel to sample identities. Specimens were delivered on dry ice to the Northwest Metabolomics Research Center and stored at − 80 °C until use.

### Quantitative lipidomics profiling

Lipid metabolite profiling was performed using the Sciex Lipidyzer platform at the Northwest Metabolomics Research Center. The Lipidyzer is a direct sample infusion-mass spectrometry (MS)-based platform that enables absolute quantification (in µM) of up to 1100 lipid species across multiple major lipid classes, including cholesterol esters (CE), diacylglycerols (DAG), triacylglycerols (TAG), free fatty acids (FFA), phosphatidylcholines (PC), phosphatidylethanolamines (PE), lysophosphatidylcholines (LPC), lysophosphatidylethanolamines (LPE), sphingomyelins (SM), ceramides (CER), dihydroceramides, hexosylceramides, and lactosylceramides. The system uses a Sciex 5500 MS instrument, a differential mobility device (SelexION), and quantitative labeled standards provided by Sciex. 54 different deuterium-labeled internal standards across the major lipid classes are included.

Frozen plasma (or ascites) specimens were thawed at room temperature for 30 min. Lipids were isolated from 25 μL of each sample using a dichloromethane/methanol extraction procedure. 25 μL plasma (or ascites), 0.45 mL of Milli-Q water, 25 μL of isotope labeled internal standard mixture, and 1.45 mL of dichloromethane/methanol (0.45:1, v:v) were added to a glass culture tube. The mixture was then vortexed for 5 s and incubated for 30 min at room temperature (25 °C). Then an additional 0.5 mL of water and 0.45 mL of dichloromethane was added and the mixture was vortexed for 5 s followed by centrifugation at 2500×*g* at 15 °C for 10 min. The lower (organic) phase was then collected and loaded into a new glass culture tube. 0.9 mL of dichloromethane was added to the original sample tubes for a second extraction after vortex mixing for 5 s and centrifugation at 2500×*g* for 10 min, and the lower organic phase was added to the first extraction. The extracts were concentrated under nitrogen, reconstituted in 250 μL of the mobile phase (10 mM ammonium acetate in DCM:MeOH (1:1, v:v), transferred to LC vials, and analyzed on the Lipidyzer using direct infusion. Rigorous quality control (QC) procedures are incorporated into standard operating protocols developed by Sciex and Metabolon to ensure high data quality and reproducibility. QC samples were included at regular intervals between study samples and used to calculate the coefficient of variation (CV) for each measured metabolite.

### Metabolite data processing

The Lipidomics Workflow Manager software provided by Sciex was used to extract MS/MS data and calculate absolute concentrations based on ratios of the metabolite peaks to relevant internal standards. Within each patient cohort, profiled analytes were excluded if (i) detectable signal was absent in > 33% of all study samples; (ii) the coefficient of variation (CV) across all QC samples exceeded 20%, or (iii) a statistically significant (P < 0.05) association was observed between concentration and specimen storage duration, among samples from control participants.

### Measurement of CA125

Plasma CA125 concentrations (U/mL) were determined using the ARCHITECT CA 125 II assay (Abbott Laboratories, Abbott Park, IL), a chemiluminescent microparticle immunoassay run on the ARCHITECT *i* System. Measurements on all study samples in both cohorts were conducted in the Department of Pathology and Laboratory Medicine at Roswell Park Comprehensive Cancer Center.

### Lipidomics statistical analyses

To assess differences in mean concentration of individual lipid species between cases and controls, linear regression was used to regress log_2_-transformed metabolite concentrations on case status (X, 0/1), with adjustment for patient age and BMI: M ~ α_0_ + α_1_ Age + α_2_ BMI + β X. Statistical significance was assessed using the P value associated with the regression β coefficient (P_β_), with correction for multiple comparisons via the Bonferroni or Benjamini–Hochberg false discovery rate (FDR) method. In subgroup analyses, early-stage (I–II) or late-stage (III–IV) cases were compared to controls. To assess differences in abundance of major lipid classes by case status, the concentrations for individual lipid species assigned to a given class were summed across all cases or all controls prior to log transformation, and linear regression was conducted as above using the resulting class-level metabolite variables. Spearman rank correlation analysis was conducted to compare levels of individual metabolites in paired ascites versus plasma specimens from a subset of women with late-stage OC in the Roswell Park patient cohort.

### Classification models

Logistic regression was used to model the log odds of case status as a linear function of log_2_ CA125, age, and BMI, with or without a given lipid metabolite (M). For each lipid species retained after QC filtering, missing values were imputed to ½ the minimum concentration detected for that species across all participants. Receiver operating characteristic (ROC) curve analyses were conducted to estimate area under the curve (AUC) and specificity at 90% sensitivity (*spec90*) of the joint model (with M) and the base model (without M). Statistical significance of the difference in *spec90* achieved by these models (Δ*spec90*) was assessed via bootstrap resampling as previously described^32^. Top-performing metabolites identified in the RP cohort were similarly assessed using data from the FH cohort. All statistical analyses were conducted using StataSE v15 (College Station, TX) and R v3.6.1.

### Differential gene expression analyses

Four independent datasets were extracted from the NIH Gene Expression Omnibus (GEO, RRID:SCR_005012) repository: GSE4122^33^, GSE57477^34^, GSE7463^35^, GSE6822^36^. First, the datasets were harmonized by mapping the identifiers to gene symbols; only genes common to all datasets were retained. Using NetworkAnalyst^37,38^, individual datasets were normalized and batch effects were adjusted using the Combat method. Differential expression analysis was performed within each dataset, comparing malignant versus benign ovarian tumor samples using *limma*, to identify differentially expressed genes and obtain their effect sizes and corresponding P values. To further control for unknown cross-study heterogeneities, random effects models based on effect sizes from individual datasets were used for meta-analysis to obtain combined effect sizes and associated P values. 106 malignant and 51 benign samples were included in total across the four studies. Analysis was restricted to 145 genes in 61 selected GO gene sets related to lipid transport.

### Ethics approval and consent to participate

The study was approved by the Institutional Review Boards at Roswell Park Comprehensive Cancer Center and Fred Hutchinson Cancer Research Center. All patients provided informed consent. The study was performed in accordance with the Declaration of Helsinki.

## Results

### Patient and tumor characteristics

Two patient cohorts were analyzed in this study, both evenly divided between women with ovarian cancer (cases) and women with benign adnexal mass (controls) (Table 1). All included participants were post-menopausal and of European ancestry (White). Cases in both cohorts had a mean age of ~ 64 years, while FH controls were somewhat older than RP controls (mean age 67.3 versus 59.5). Mean BMI was modestly reduced in cases relative to controls in both cohorts, while BMI distributions were slightly higher in RP versus FH study participants. All malignant and benign tumors in the FH cohort were classified as serous histology, and 84% of cases were late-stage (III-IV); by contrast, serous tumors accounted for 53% of cases and 33% of controls among RP patients, and 57% of cases were late-stage (Table S1).

**Table 1:** Study participant and tumor characteristics.

	Fred Hutch (FH)				Roswell Park (RP)			
	Cases		Controls		Cases		Controls	
	n = 50		n = 50		n = 60		n = 58	
	n	%	n	%	n	%	n	%
**Age**								
< 50	0	0.0	0	0.0	0	0.0	8	13.8
50–59	16	32.0	9	18.0	21	35.0	22	37.9
60–69	23	46.0	23	46.0	25	41.7	19	32.8
70+	11	22.0	18	36.0	14	23.3	9	15.5
Mean (sd)	64.3 (7.8)		67.3 (8.1)		64.3 (8.4)		59.5 (9.8)	
**BMI**								
< 25	21	44.7	17	36.2	11	18.3	14	24.1
25–29.9	11	23.4	8	17.0	21	35.0	12	20.7
30+	15	31.9	22	46.8	28	46.7	32	55.2
Mean (sd)	27.4 (5.7)		29.2 (8.1)		29.8 (6.1)		32.4 (8.3)	
**Histology**								
Serous	50	100.0	50	100.0	32	53.3	19	32.8
Non-serous	0	0.0	0.0	0.0	28	46.7	39	67.2
**Stage**								
I	5	10.0			16	26.7		
II	3	6.0			10	16.7		
III	39	78.0			18	30.0		
IV	3	6.0			16	26.7		

### Lipidome profiles differ by case status

**Figure 1:**
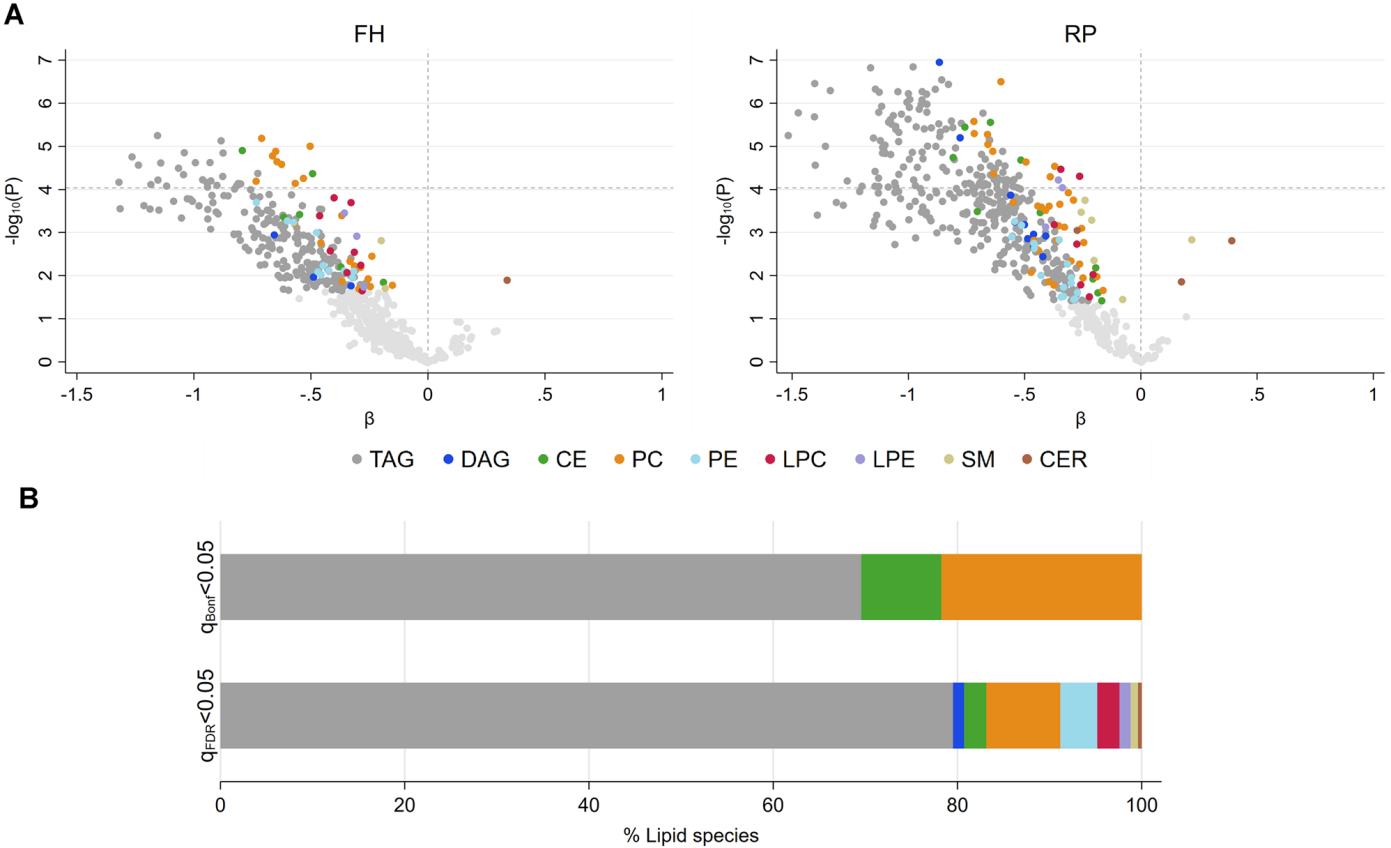
Plasma lipid metabolite differences between cases and controls in Fred Hutch (FH) and Roswell Park (RP) patient cohorts. Log_2_ metabolite concentrations were regressed on case status with adjustment for age and BMI. (**A**) Volcano plots depicting estimated beta (β) coefficients and − log_10_ P values for 546 individual lipid metabolites (left: FH, right: RP). Species satisfying FDR q < 0.05 are color-coded by lipid class, as indicated, with the remainder shown in light gray. Dashed horizontal line marks the Bonferroni-corrected threshold for statistical significance: − log_10_ (0.05/546) = 4.04. (**B**) Lipid class assignments of species reaching q_Bonf_ < 0.05 (n = 23, top), or q_FDR_ < 0.05 (n = 249, bottom), in both cohorts.

Quantitative global lipidomics profiling using the Sciex Lipidyzer platform enabled ascertainment of absolute concentrations (µM) for a large number of plasma lipid metabolites, with high reproducibility, sensitivity, and accuracy. 546 species assigned to ten major lipid classes were retained for analysis after imposing quality-control filters (Table S2, Fig. S1). The mean CV for all included metabolites measured in the pooled QC replicate samples was < 7.6% (Fig. S2). Mean concentrations of individual lipid species across all study participants ranged from < 0.2 to > 1900 µM (Table S3). Comparison of mean metabolite concentrations in cases versus controls, after adjustment for age and BMI, revealed similar global patterns of alterations in both patient cohorts, with the vast majority of species exhibiting reduced abundance in cases (Fig. 1). Using the false discovery rate (FDR < 0.05) to account for multiple comparisons, 253 species differed by case status in the FH cohort, and 249 were validated in the RP cohort. Using the highly stringent Bonferroni threshold (P < 0.05/546 = 9.16 × 10^−5^), 36 species differed by case status in the FH cohort, and 23 were validated in the RP cohort. The most robust differences observed, based on smallest P values, included reductions in TAG, PC, and CE species (Table S4). Significant decreases were also found for DAG, PE, LPC, LPE, and SM species. Fold changes in mean concentration ranged from 0.35 to 0.87. A single ceramide species, CER(18:0), was elevated (1.3-fold) in cases vs. controls (FDR < 0.05), in both cohorts (Fig. 1).

### Lipid metabolite alterations vary by disease stage

**Figure 2:**
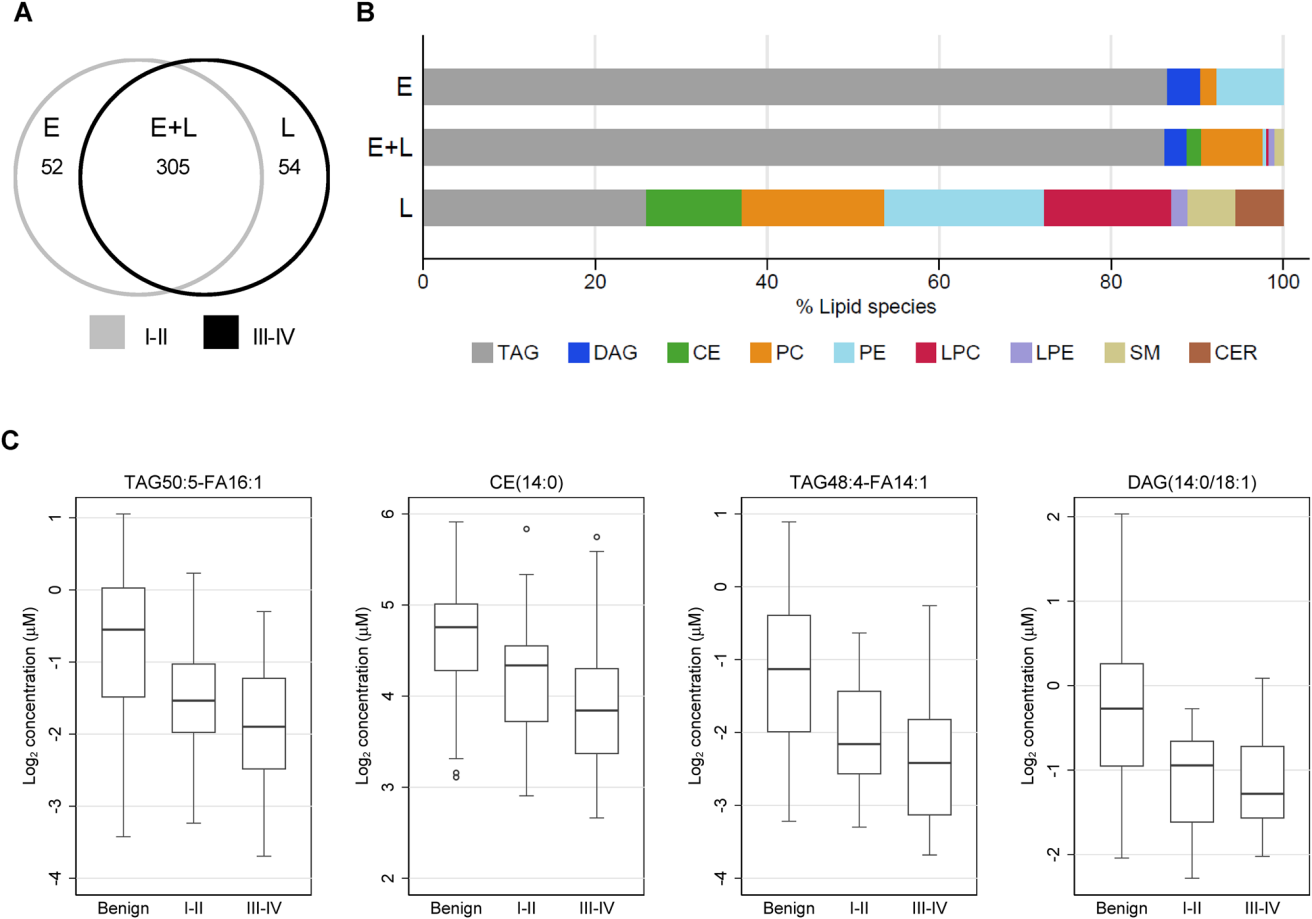
Plasma lipid metabolite differences in early (I–II)-stage or late (III–IV)-stage cases versus controls. Log_2_ metabolite concentrations in the RP cohort were regressed on case status with adjustment for age and BMI. (**A**) 411 lipid species exhibited significant (FDR q < 0.05) differences in either early-stage or late-stage cases versus controls, including species significant in only the early-stage subgroup (E: n = 52), only the late-stage subgroup (L: n = 54), or both subgroups (E + L: n = 305). (**B**) Distributions of class assignments for lipid species in each category (E, E + L, L). (**C**) Box plots of plasma concentrations for top trending lipid metabolites (P_trend_ < 9 × 10^−7^).

To investigate whether the circulating lipidome exhibited distinct alterations in patients with early-stage (I–II) or late-stage (III-IV) cancers versus benign tumors, we conducted stage-stratified analyses using data from the RP cohort, in which ~ 43% of cases had early-stage disease (vs. only 16% of cases in FH cohort). 74% of lipid species altered in either early- or late-stage cancers (at FDR < 0.05) were common to both subgroups, and directionally concordant, while smaller subsets exhibited significant changes only in women with early- or late-stage tumors (Fig. 2A). Metabolites identified as differentially abundant only in early-stage patients included species in four lipid classes (TAG, DAG, PC, PE), while those differing only in late-stage patients included a greater diversity of species across 8 distinct lipid classes (TAG, CE, PC, PE, LPC, LPE, SM, CER) (Fig. 2B). Multiple lipid species exhibited statistically significant linear trends in plasma concentration when comparing across patients with benign disease, early-stage OC, and late-stage OC. Box plots for the four species with the strongest linear trends (P_trend_ < 9 × 10^–7^) are shown in Fig. 2C. Among 100 metabolites satisfying P < 9.16 × 10^–5^, 99 decreased in mean concentration with increasing disease severity, including a number of glycerolipids (TAG/DAG), phospholipids (PC), lysophospholipids (LPC/LPE), and CE species. Only CER(18:0) showed a positive linear trend from benign through early- and late-stage disease. The top-ranked metabolites in stage-stratified analyses are listed in Table S5.

### Specific lipid classes differ by case status and disease stage

In addition to analyzing individual lipid species, we also examined the summed concentrations of all species assigned to specific lipid classes. Using the Bonferroni significance threshold (0.05/10 = 0.005), three lipid classes—PE, LPC, LPE—exhibited significant alterations (reductions) in cases versus controls in the FH patient cohort, and all three classes were validated in the RP cohort (Fig. S3A). In stage-stratified analyses, certain significant class-level differences were detected only in late-stage cases (LPC, LPE, SM), only in early-stage (DAG, TAG), or in both subgroups (PC, PE) (Fig. S3B).

### Classifying adnexal masses using plasma CA125 and lipid metabolites

**Figure 3:**
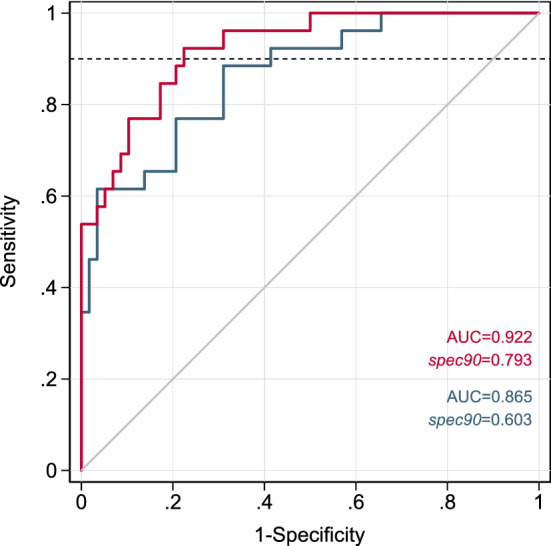
Classification performance of joint model comprised of DAG(16:1/18:1), CA125, age, and BMI, versus base model comprised of CA125, age, and BMI only. ROC curve analysis conducted using RP cohort data from early-stage (I–II) cases and controls. Joint model (red), base model (blue).

To determine whether circulating lipid metabolites may aid in the identification and clinical triage of women with malignant versus benign adnexal masses that meet clinical criteria for surgical excision, we evaluated the ability of single lipid species to enhance the diagnostic classification accuracy of an established protein biomarker, CA125, in combination with age and BMI. The distribution of plasma CA125 in cases and controls, by cohort, is shown in Fig. S4. Late-stage cases had significantly higher mean CA125 levels in both sample sets; CA125 levels in early-stage cases were similar to (FH) or higher than (RP) levels in controls. Assessment of the classification performance of a ‘base model’ comprised of CA125, age, and BMI, underscored the limited ability of CA125 to accurately identify early-stage cancers, in particular, as compared to late-stage malignancies where CA125 performs reasonably well (Fig. S5). Thus, our candidate marker screening efforts focused on lipid species with potential to improve discrimination between early-stage cases and benign controls. Given that a clinical triage test for women with an adnexal mass requires high sensitivity to ensure patients with cancer are appropriately identified and referred to specialty care, we selected specificity at 90% sensitivity (*spec90*) as a clinically-relevant performance metric for evaluation. Using data from the RP cohort, we identified a number of individual lipid species which, when combined with CA125, age, and BMI, outperformed the base model and yielded improvements in *spec90* of up to ~ 20% (Table 2). The joint model using one such metabolite, DAG(16:1/18:1), achieved a *spec90* of almost 80% (Fig. 3), with the observed boost above the base model reaching statistical significance (P = 0.04). While only a small number of FH participants had early-stage disease, similar assessment of ten leading candidate markers using this independent dataset yielded consistent improvements in *spec90* for three species, including DAG(16:1/18:1) (Table S6).

**Table 2:** Performance characteristics of single lipid species combined with CA125, age, and BMI, in classifying early-stage (I-II) cases versus controls.

	Class	Metabolite	*spec90*	*Δspec90*	P_Δs_	AUC	ΔAUC	P_ΔA_
1	DAG	DAG(16:1/18:1)	0.79	0.19	0.04	0.92	0.06	0.02
2	CE	CE(18:1)	0.67	0.07	0.08	0.87	0.00	0.38
3	CE	CE(17:0)	0.69	0.09	0.11	0.88	0.01	0.31
4	DAG	DAG(20:0/20:0)	0.71	0.10	0.13	0.88	0.01	0.26
5	TAG	TAG52:2-FA20:2	0.81	0.21	0.17	0.91	0.05	0.06
6	TAG	TAG54:3-FA20:3	0.79	0.19	0.20	0.91	0.04	0.08
7	PE	PE(P-16:0/20:3)	0.74	0.14	0.20	0.88	0.01	0.32
8	DAG	DAG(18:0/18:2)	0.76	0.16	0.22	0.90	0.03	0.10
9	TAG	TAG56:4-FA18:0	0.81	0.21	0.23	0.90	0.04	0.12
10	CE	CE(20:0)	0.67	0.07	0.23	0.87	0.01	0.38

Using data from the RP cohort, logistic regression was used to model the log odds of case status as a linear function of log_2_ CA125, age, and BMI, with or without a given lipid metabolite (M), after imputing missing values for each species to ½ the minimum concentration detected for that species across all participants. Receiver operating characteristic (ROC) curve analysis was conducted to estimate area under the curve (AUC) and specificity at 90% sensitivity (*spec90*) conferred by the joint model (with M) versus the base model (without M: AUC = 0.865, *spec90* = 0.603). Lipids with Δ*spec90* > 0.05 were selected and ranked by corresponding P value (P_Δs_). Performance metrics listed for top 10 lipid species.

### Correlation of lipid concentrations in plasma and ascites

To assess the relationship between lipid metabolite levels in the tumor microenvironment versus circulation, we performed Spearman rank correlation analyses using lipid measurements in paired ascites and plasma samples from a subset of 15 patients with late-stage OC. We found evidence of moderate to weak correlation (0.2 ≥|rho|< 0.6) across compartments for 240 of > 500 lipid species evaluated; most correlations (> 80%) were positive (Fig. S6). All 16 of the lipids which satisfied P_rho_ < 0.05 were TAGs (with rho > 0.5). 15 were also significantly reduced in OC cases versus benign controls (Fig. 1, FDR < 0.05), and three were validated at high stringency (Bonferroni q < 0.05): TAG48:2-FA14:0, TAG48:3-FA16:0, TAG48:3-FA18:2. Ascites-plasma correlations were not statistically significant after accounting for multiple testing.

### Lipid transport-related gene expression changes in malignant versus benign ovarian tumors

**Figure 4:**
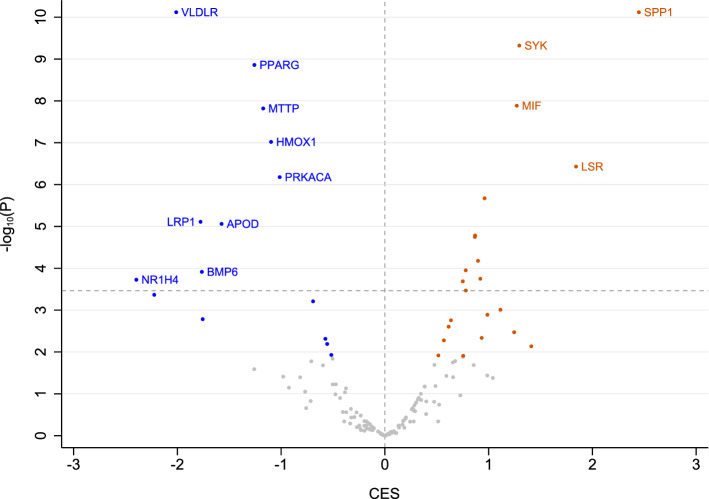
Gene expression differences in malignant versus benign ovarian tumor tissues. Differential expression analysis of 145 selected lipid-transport-related genes was conducted using RNA profiling data extracted from the NIH GEO archive. Results obtained from four independent sample sets (total N = 106 malignant, N = 51 benign) were meta-analyzed using NetworkAnalyst. Combined effect sizes (CES) and − log_10_ P values are plotted. Genes satisfying FDR q < 0.05 are colored blue (downregulated) or orange (upregulated). Dashed horizontal line, Bonferroni-corrected threshold for statistical significance (− log_10_ (0.05/145) = 3.59).

Linking alterations in the plasma metabolome to metabolic and biological changes in diseased tissues remains an active area of investigation. To further explore such relationships, we sought insight into specific molecular features that differentiate ovarian cancers from benign ovarian tumors, using publicly-available gene expression profiling datasets in the NIH GEO archive (Table S7). Building on recent evidence pointing to key roles for increased lipid uptake in ovarian malignancies^8,10,11^, we focused our differential expression analysis on ~ 150 genes related to lipid transport (Table S8). Our meta-analysis of four independent datasets encompassing 106 malignant and 51 benign ovarian samples revealed 37 genes differentially expressed by case group at FDR < 0.05. 13 genes remained significant after Bonferroni correction and showed two-fold or greater fold changes (Fig. 4). Notably, among the largest expression changes observed were for two genes encoding cell-surface lipoprotein receptors, *LSR* (lipolysis stimulated receptor) and *VLDLR* (very low density lipoprotein receptor). While *LSR* expression was elevated in malignant versus benign tumors, *VLDLR* expression was reduced. These intriguing findings indicate that expression of specific lipid transporter genes represents a distinguishing feature of ovarian cancers relative to benign tumors, and further suggest that tumor biological differences in lipid uptake may underlie observed alterations in plasma lipid profiles in women with these distinct clinical conditions.

## Discussion

Alterations in lipid metabolism have been reported in many human cancers, including OC, and are thought largely to reflect the increased metabolic demands associated with rapid cell proliferation^6^. Improved molecular understanding of lipid metabolic phenotypes is anticipated to generate new approaches for detecting, monitoring, and intercepting cancer pathogenesis. Drawing on extensive resources from two major cancer centers in the U.S., we conducted quantitative lipidomics profiling of 218 plasma specimens isolated from women diagnosed with ovarian cancer or benign adnexal mass, with the goal of comprehensively characterizing systemic lipid alterations across this disease spectrum and identifying specific metabolites which can help differentiate patients with OC versus benign pathology. Integration of plasma lipidomics profiles with paired measurements in ascites and with additional gene expression analyses in ovarian tumor tissues enabled multi-level assessments, providing new biological and molecular insights into the lipid metabolic phenotypes that distinguish malignant from benign ovarian pathology.

Distinguishing between malignant and benign adnexal masses remains an important clinical challenge with significant implications for patient prognosis and survival^39^. Women with OC who are referred to tertiary care specialists have increased survival relative to those treated by general gynecologists/surgeons, likely due to optimal surgical debulking and clinical management^40^. Treatment of women with benign masses by specialists is unlikely to confer medical benefit or harm, but imposes a needless burden on the healthcare system and may increase patient anxiety. Clinical triage tests that prioritize high sensitivity for cancer detection, and secondarily maximize specificity, can help ensure that OC patients receive optimal care while minimizing wasteful utilization and reducing anxiety among women without cancer^41^. While existing tests based on CA125, alone or in combination with other circulating protein markers, perform relatively well in separating late-stage OC from benign disease, accurate identification of early-stage cancers among women presenting with a pelvic mass remains challenging^42–44^. Our modeling results presented herein suggest that combining CA125 with specific individual lipid metabolites, such as DAG(16:1/18:1), may provide a substantial boost to specificity at 90% sensitivity, relative to CA125 alone, in separating early-stage ovarian malignancies from benign adnexal masses. Further performance gains may be feasible through construction of models that integrate candidate lipid metabolite markers with additional protein markers included in emerging clinical triage tools—HE4, prealbumin, transferrin, β2 microglobulin, apolipoprotein AI^45,46^. Recent evidence from prospective cohort studies suggests that circulating lipid alterations may also be detectable in pre-diagnostic specimens collected from women who later develop OC^47–49^.

Salient differences in plasma metabolite profiles between cancer cases and controls included wide-scale reductions in glycerolipids, glycerophospholipids, and cholesterol esters, coupled with isolated elevation of a single CER species (CER(18:0)). The 249 individual lipid metabolites that differed in concentration by case status at FDR < 0.05, in both patient cohorts, included 30 glycerophospholipids, nine lysophospholipids, six cholesterol esters, small numbers of diacylglycerols or sphingomyelins, one ceramide, and nearly 200 triacylglycerols. While changes in certain lipids reached significance (FDR < 0.05) only in patients with early- or late-stage cancer, apparent stage specificity should be interpreted cautiously, as subgroup sample sizes were limited; non-significant point estimates for 105 of 106 such species were directionally concordant with (though weaker in magnitude than) those observed in the other subgroup. Significant linear trends in concentration were noted for many lipids across patients with benign disease through early- and late-stage OC.

Circulating metabolite alterations identified in this report confirm key overall patterns described in our initial analysis of FH cohort participants^23^, while significantly expanding the scope of lipidome interrogation and spectrum of included patients with adnexal masses. In comparison to the first-generation untargeted lipidomics platform we employed previously, the Sciex Lipidyzer system enabled absolute quantitation using labeled internal standards and provided superior reproducibility and sensitivity. Since 2015, subsequent independent studies conducted in Scandinavia, Europe, and China have provided further support for wide-scale reductions in the levels of many circulating phospholipids and glycerolipids in women with OC versus benign disease^24,26–29^. Elevations in CER(18:0) have also been reported^28,29,50^. In minor contrast to our results, Hilvo et al. found *increased* levels of small numbers of long-chain TAGs^29^. While several TAGs with ≥ 56 total carbons did have positive beta coefficients in our regression analyses, none of these suggested differences reached statistical significance at P < 0.05. The strong similarity of metabolite alterations by case status observed in both of our patient cohorts, notwithstanding the histologic diversity of masses in the RP cohort, suggests that metabolic profiles associated with malignancy versus non-malignancy may supersede possible histotype-specific differences. Similar conclusions were reached in a recent study from Finland^29^, but larger samples of non-serous patients are needed for definitive confirmation.

The biological basis for wide-ranging reductions in circulating glycerolipids, glycero-phospholipids, and cholesterol esters in women with OC versus benign disease remains an area of active investigation. Consistent with a recent report focusing on OC survival and prognosis^27^, we found that concentrations of multiple lipid species in plasma and ascites were positively correlated (lower levels in ascites tracked with lower levels in blood). Since the composition of ascites fluid is thought to represent the OC tumor microenvironment, observed correlations between ascites and blood lipid levels may reflect increased lipid uptake and consumption by growing tumors^27^, key functional features of OC described in past studies^8,10^. Lipids represent an important energy source to support rapid tumor proliferation and expansion, and the observed widescale reduction in the plasma lipidome represents a plausible systemic readout of this malignancy. Interestingly, recent studies of pre-diagnostic blood samples suggest that certain metabolites identified in our analysis as differentially abundant at the time of diagnosis between women with OC versus benign disease, may also exhibit changes in circulation years before an OC diagnosis. These include PCs (34:2, 38:3), PEs (36:3, 38:5), TAGs (46:0, 52:4/5, 54:4/5/6/7, 56:4/5/7/8), and SM 22:1.

Our analyses of lipid-transport-related gene expression profiles were aimed at further defining the molecular characteristics differentiating malignant versus benign ovarian tumors. Intriguingly, while expression of two lipoprotein receptor genes (*VLDLR*, *LRP1*) was *reduced* in OC by ~ fourfold, expression of a third receptor gene, *LSR*, was *elevated* by a similar magnitude. In a recent proteomics screen, Hiramatsu et al. identified *LSR* as a novel OC tumor antigen and reported intensely elevated LSR protein expression in OC tissues versus normal ovaries^11^. The authors provided further evidence of LSR-mediated VLDL uptake; LSR-dependent cell proliferation in response to VLDL; anti-tumor activity from LSR blockade; and an association between high LSR and poor prognosis in OC patients. Increased levels of circulating triglyceride-rich lipoproteins and LDL have been reported in LSR-deficient mice^51^, suggesting that LSR is important for internalization of these lipoproteins, and cannot be fully compensated for by other lipoprotein receptors (e.g., VLDLR, LDLR, LRP1). Elevated LSR expression has also been identified in colon cancer and found to correlate with poor prognosis^52^. Our own results extend these findings and indicate that specific patterns of lipoprotein gene expression (LSR-high/VLDLR-low/LRP1-low) are a distinguishing feature of malignant versus benign ovarian tumors. Integrating results across our tumor gene expression analysis and plasma/ascites lipidomics profiling studies provides support for a model of altered lipoprotein uptake in OC relative to benign tumors, mediated in part through the membrane receptor LSR (Fig. S7).

Strengths of this study included use of the Sciex Lipidyzer global lipidomics profiling system, which enabled extensive coverage of the plasma lipidome and absolute quantitation of metabolite concentrations with high sensitivity, reproducibility, and accuracy. The mean concentrations of 546 analyzed lipid metabolites spanned four orders of magnitude (from ~ 0.2 to ~ 2000 µM). Mean CV values by lipid class ranged from 3.8 to 14.2%, and > 200 individual lipid species had CVs < 6%. Our large overall sample size (n = 218) and inclusion of two independent patient cohorts from distinct clinical sites provided us with a robust study design, reducing the likelihood of chance findings. Statistical adjustment for age and BMI helped mitigate the influence of potential confounders. Our inclusion of both serous and non-serous ovarian pathologies in the RP cohort allowed for lipidomic measurements across a broader clinical spectrum of patients who present with adnexal masses. Collection, processing, and storage of blood specimens at each site were conducted according to standardized protocols. Plasma CA125 was quantitated using a single clinical-grade assay performed in one facility.

Our study had certain limitations. While the Sciex Lipidizer profiling system enabled measurement of absolute concentrations of nearly 550 plasma metabolites across ten lipid classes, certain classes were either not represented (e.g., eicosanoids and other oxylipins) or represented by a relatively small pool of individual lipid species (e.g., LPE, FFA, CER). A number of detectable species were excluded from analysis after imposition of quality control filters, particularly percent missingness among study samples or CV among replicates. Investigations using next-generation lipidomics platforms with even higher sensitivity (e.g., liquid chromatography electrospray ionization tandem mass spectrometry, LC/ESI–MS/MS) and more extensive coverage may help determine the extent to which additional lipid metabolite signals were missed. We note that blood lipid levels, particularly triglycerides, can be affected by fasting status. While blood draws in the FH cohort were performed after a 12-h fast, collections in the RP cohort occurred under non-fasting conditions. Nevertheless, within each cohort, specimens from cases and controls were collected in a uniform manner, and metabolite differences ultimately identified between case groups were very similar in both cohorts. Our study included a limited number of stage I–II cancers, reflecting in part the underlying stage distribution of diagnosed OC cases; our statistical power was thus constrained when screening for and assessing the performance of novel candidate lipid metabolite markers for localized disease. Promising lead candidates we identified (e.g., DAG(16:1/18:1)) warrant further confirmation in larger study populations enriched for early-stage and pre-menopausal cases.

This study provides further insight into systemic lipid metabolic phenotypes that distinguish women with ovarian cancer from those with benign adnexal mass. Through integration of quantitative global lipidomics profiling in plasma and ascites and differential gene expression analysis in tissue, our results reveal novel relationships between tumor lipoprotein receptor expression patterns, blood lipid metabolite profiles, and malignant versus benign ovarian tumor pathology. These findings are consistent with growing evidence pointing to an important role for lipid uptake pathways in OC pathogenesis, and further suggest the potential translational utility of specific circulating lipid metabolites to aid in the clinical diagnosis and triage of women with adnexal mass.

## Supplementary Information

Supplementary Information.

## Data Availability

The data sets generated or analysed during the current study are available for non-commercial use from the corresponding authors upon reasonable request.
