# Supplementary material for: Quantitative global lipidomics analysis of patients with ovarian cancer versus benign adnexal mass
Source: Sci Rep. 2021 Sep 13;11:18156. doi: 10.1038/s41598-021-97433-x (PMC8438087; doi:10.1038/s41598-021-97433-x)

SUPPLEMENTAL MATERIAL

Table S1. Histologic classifications of malignant and benign tumors in FH and RP cohorts.

Table S2. Lipid species retained for analysis in both patient cohorts.

Table S3. Plasma concentrations (μM) for representative lipid species by class.

Table S4. Selected top differential metabolites by case status.

Table S5. Selected top differential metabolites by case status in stage-stratified analyses.

Table S6. Performance characteristics for selected lipid species (Table 2) using data from FH cohort.

Table S7. Patient and tumor characteristics (GEO samples).

Table S8. Lipid transport-related GO gene sets selected and individual genes available for analysis.

Figure S1. Lipid class assignments for 546 analyzed lipid species.

Figure S2. Distributions of coefficients of variation (CV) calculated for 546 analyzed lipid species.

Figure S3. Lipid class differences in cases versus controls.

Figure S4. Plasma CA125 values (U / mL) for cases and controls.

Figure S5. ROC curves and performance metrics for base models comprised of CA125, age, and BMI.

Figure S6. Spearman correlation analysis of lipid concentrations in paired ascites and plasma.

Figure S7. Schematic summary.

Table S1. Histologic classifications of malignant and benign tumors in FH and RP cohorts.

FRED HUTCH

Cases [50]			Controls [50]		
**SEROUS [50]**	**n**	**%**	**SEROUS [50]**	**n**	**%**
Serous carcinoma	47	94.0	Serous cystadenofibroma	19	38.0
STIC lesion	1	2.0	Serous cystadenoma	14	28.0
Other	2	4.0	Serous adenofibroma	4	8.0
			Other	13	26.0

ROSWELL PARK

Cases [60]			Controls [58]		
**SEROUS [32]**	**n**	**%**	**SEROUS [19]**	**n**	**%**
Cystadenofibroma, malignant serous	1	3.1	Serous adenoma	1	5.3
Papillary serous adenocarcinoma	4	12.5	Serous cyst	3	15.8
Papillary serous cystadenocarcinoma	1	3.1	Serous cystadenofibroma	4	21.1
Serous adenocarcinoma, NOS	4	12.5	Serous cystadenoma	11	57.9
Serous carcinoma, NOS	13	40.6			
Serous cystadenocarcinoma, NOS	7	21.9			
Serous surface papillary carcinoma	2	6.3			
**NON-SEROUS [28]**	**n**	**%**	**NON-SEROUS [39]**	**n**	**%**
Carcinosarcoma, NOS	2	7.1	Benign Teratoma	1	2.6
Clear cell adenocarcinoma, NOS	1	3.6	Brenner Tumor	1	2.6
Clear cell carcinoma	1	3.6	Complex Cyst	1	2.6
Endometrioid adenocarcinoma, NOS	3	10.7	Corpus Luteum Cyst	1	2.6
Leiomyosarcoma, NOS	1	3.6	Cyst	2	5.1
Lymphoma, malig, large B-cell, diffuse, NOS	1	3.6	Cystadenoma	5	12.8
Malignant melanoma, NOS	1	3.6	Endometriosis of Ovary	1	2.6
Mixed cell adenocarcinoma	5	17.9	Endometroid cystadenofibroma	1	2.6
Mucinous adenocarcinoma	6	21.4	Endosalpingiosis	1	2.6
Mucinous carcinoma	1	3.6	Epithelial inclusion cyst	1	2.6
Mucinous cystadenocarcinoma, NOS	3	10.7	Fibroma	2	5.1
Mullerian mixed tumor	1	3.6	Fibrothecoma	1	2.6
Papillary adenocarcinoma, NOS	1	3.6	Follicular Cyst	2	5.1
Yolk sac tumor	1	3.6	Hemorrhagic Corpus Luteal Cyst	2	5.1
			Hemorrhagic Cyst	1	2.6
			Hydrosalpinx	1	2.6
			Mature Cystic Teratoma	2	5.1
			Mesothelial Inclusion Cyst	1	2.6
			Mucinous Adenoma	2	5.1
			Mucinous Cystadenoma	7	18.0
			Simple Cyst	2	5.1
			Struma Ovarii	1	2.6

Table S2. Lipid species retained for analysis in both patient cohorts.

	Metabolite	Class
1	CE(12:0)	CE
2	CE(14:0)	CE
3	CE(14:1)	CE
4	CE(15:0)	CE
5	CE(16:1)	CE
6	CE(17:0)	CE
7	CE(18:0)	CE
8	CE(18:1)	CE
9	CE(18:2)	CE
10	CE(18:3)	CE
11	CE(18:4)	CE
12	CE(20:0)	CE
13	CE(20:1)	CE
14	CE(20:2)	CE
15	CE(20:3)	CE
16	CE(20:5)	CE
17	CE(22:2)	CE
18	CE(22:4)	CE
19	CE(22:5)	CE
20	CE(22:6)	CE
21	CER(18:0)	CER
22	CER(24:0)	CER
23	CER(24:1)	CER
24	HCER(22:0)	CER
25	HCER(24:0)	CER
26	LCER(24:1)	CER
27	DAG(14:0/18:1)	DAG
28	DAG(14:1/18:1)	DAG
29	DAG(16:0/16:0)	DAG
30	DAG(16:0/16:1)	DAG
31	DAG(16:0/18:1)	DAG
32	DAG(16:0/18:2)	DAG
33	DAG(16:1/18:1)	DAG
34	DAG(16:1/18:2)	DAG
35	DAG(18:0/18:1)	DAG
36	DAG(18:0/18:2)	DAG
37	DAG(18:1/18:1)	DAG
38	DAG(18:1/18:2)	DAG
39	DAG(18:1/20:3)	DAG
40	DAG(18:1/20:4)	DAG
41	DAG(18:1/22:6)	DAG
42	DAG(20:0/20:0)	DAG
43	FFA(17:0)	FFA
44	FFA(20:5)	FFA
45	FFA(22:6)	FFA
46	FFA(24:0)	FFA
47	LPC(15:0)	LPC
48	LPC(16:0)	LPC
49	LPC(17:0)	LPC
50	LPC(18:0)	LPC
51	LPC(18:1)	LPC
52	LPC(18:2)	LPC
53	LPC(20:2)	LPC
54	LPC(20:3)	LPC
55	LPC(20:4)	LPC
56	LPC(22:5)	LPC
57	LPC(22:6)	LPC
58	LPE(16:0)	LPE
59	LPE(18:0)	LPE
60	LPE(18:1)	LPE
61	PC(14:0/18:1)	PC
62	PC(14:0/18:2)	PC
63	PC(14:0/20:3)	PC
64	PC(14:0/20:4)	PC
65	PC(15:0/18:1)	PC
66	PC(15:0/18:2)	PC
67	PC(16:0/14:0)	PC
68	PC(16:0/16:1)	PC
69	PC(16:0/18:0)	PC
70	PC(16:0/18:1)	PC
71	PC(16:0/18:2)	PC
72	PC(16:0/18:3)	PC
73	PC(16:0/20:2)	PC
74	PC(16:0/20:3)	PC
75	PC(16:0/20:4)	PC

	Metaboltie	Class
76	PC(16:0/20:5)	PC
77	PC(16:0/22:4)	PC
78	PC(16:0/22:5)	PC
79	PC(16:0/22:6)	PC
80	PC(17:0/18:1)	PC
81	PC(17:0/18:2)	PC
82	PC(17:0/20:4)	PC
83	PC(18:0/16:1)	PC
84	PC(18:0/18:0)	PC
85	PC(18:0/18:1)	PC
86	PC(18:0/18:2)	PC
87	PC(18:0/18:3)	PC
88	PC(18:0/20:3)	PC
89	PC(18:0/20:4)	PC
90	PC(18:0/20:5)	PC
91	PC(18:0/22:4)	PC
92	PC(18:0/22:5)	PC
93	PC(18:0/22:6)	PC
94	PC(18:1/16:1)	PC
95	PC(18:1/18:1)	PC
96	PC(18:1/18:2)	PC
97	PC(18:1/18:3)	PC
98	PC(18:1/20:2)	PC
99	PC(18:1/20:3)	PC
100	PC(18:1/20:4)	PC
101	PC(18:1/20:5)	PC
102	PC(18:1/22:5)	PC
103	PC(18:1/22:6)	PC
104	PC(18:2/16:1)	PC
105	PC(18:2/18:2)	PC
106	PC(18:2/20:3)	PC
107	PC(18:2/20:4)	PC
108	PE(16:0/18:1)	PE
109	PE(16:0/18:2)	PE
110	PE(16:0/20:4)	PE
111	PE(16:0/22:6)	PE
112	PE(18:0/18:0)	PE
113	PE(18:1/18:1)	PE
114	PE(18:1/18:2)	PE
115	PE(18:1/20:4)	PE
116	PE(18:2/16:1)	PE
117	PE(O-16:0/20:4)	PE
118	PE(P-16:0/18:2)	PE
119	PE(P-16:0/20:3)	PE
120	PE(P-16:0/22:6)	PE
121	PE(P-18:0/18:1)	PE
122	PE(P-18:0/18:2)	PE
123	PE(P-18:0/20:3)	PE
124	PE(P-18:0/22:6)	PE
125	PE(P-18:1/18:1)	PE
126	PE(P-18:1/18:2)	PE
127	PE(P-18:1/20:3)	PE
128	PE(P-18:1/20:4)	PE
129	PE(P-18:1/22:6)	PE
130	SM(14:0)	SM
131	SM(18:1)	SM
132	SM(20:1)	SM
133	SM(22:0)	SM
134	SM(22:1)	SM
135	SM(24:0)	SM
136	SM(24:1)	SM
137	SM(26:0)	SM
138	SM(26:1)	SM
139	TAG44:0-FA16:0	TAG
140	TAG44:1-FA16:0	TAG
141	TAG44:1-FA18:1	TAG
142	TAG44:2-FA16:0	TAG
143	TAG44:2-FA18:2	TAG
144	TAG45:1-FA16:0	TAG
145	TAG46:0-FA14:0	TAG
146	TAG46:0-FA16:0	TAG
147	TAG46:1-FA12:0	TAG
148	TAG46:1-FA14:0	TAG
149	TAG46:1-FA14:1	TAG
150	TAG46:1-FA16:0	TAG

	Metabolite	Class
151	TAG46:1-FA16:1	TAG
152	TAG46:1-FA18:1	TAG
153	TAG46:2-FA12:0	TAG
154	TAG46:2-FA14:0	TAG
155	TAG46:2-FA14:1	TAG
156	TAG46:2-FA16:0	TAG
157	TAG46:2-FA16:1	TAG
158	TAG46:2-FA18:1	TAG
159	TAG46:2-FA18:2	TAG
160	TAG46:3-FA16:0	TAG
161	TAG46:3-FA16:1	TAG
162	TAG46:3-FA18:1	TAG
163	TAG46:3-FA18:2	TAG
164	TAG46:4-FA18:2	TAG
165	TAG47:0-FA15:0	TAG
166	TAG47:0-FA16:0	TAG
167	TAG47:1-FA15:0	TAG
168	TAG47:1-FA16:0	TAG
169	TAG47:1-FA16:1	TAG
170	TAG47:1-FA18:1	TAG
171	TAG47:2-FA18:1	TAG
172	TAG47:2-FA18:2	TAG
173	TAG48:0-FA14:0	TAG
174	TAG48:0-FA16:0	TAG
175	TAG48:0-FA18:0	TAG
176	TAG48:1-FA12:0	TAG
177	TAG48:1-FA14:0	TAG
178	TAG48:1-FA14:1	TAG
179	TAG48:1-FA16:0	TAG
180	TAG48:1-FA16:1	TAG
181	TAG48:1-FA18:0	TAG
182	TAG48:1-FA18:1	TAG
183	TAG48:2-FA12:0	TAG
184	TAG48:2-FA14:0	TAG
185	TAG48:2-FA14:1	TAG
186	TAG48:2-FA16:0	TAG
187	TAG48:2-FA16:1	TAG
188	TAG48:2-FA18:0	TAG
189	TAG48:2-FA18:1	TAG
190	TAG48:2-FA18:2	TAG
191	TAG48:3-FA12:0	TAG
192	TAG48:3-FA14:0	TAG
193	TAG48:3-FA14:1	TAG
194	TAG48:3-FA16:0	TAG
195	TAG48:3-FA16:1	TAG
196	TAG48:3-FA18:1	TAG
197	TAG48:3-FA18:2	TAG
198	TAG48:3-FA18:3	TAG
199	TAG48:4-FA12:0	TAG
200	TAG48:4-FA14:0	TAG
201	TAG48:4-FA14:1	TAG
202	TAG48:4-FA16:0	TAG
203	TAG48:4-FA16:1	TAG
204	TAG48:4-FA18:1	TAG
205	TAG48:4-FA18:2	TAG
206	TAG48:4-FA18:3	TAG
207	TAG48:5-FA18:2	TAG
208	TAG49:0-FA15:0	TAG
209	TAG49:0-FA16:0	TAG
210	TAG49:0-FA17:0	TAG
211	TAG49:0-FA18:0	TAG
212	TAG49:1-FA14:0	TAG
213	TAG49:1-FA15:0	TAG
214	TAG49:1-FA16:0	TAG
215	TAG49:1-FA16:1	TAG
216	TAG49:1-FA17:0	TAG
217	TAG49:1-FA18:1	TAG
218	TAG49:2-FA14:0	TAG
219	TAG49:2-FA15:0	TAG
220	TAG49:2-FA16:0	TAG
221	TAG49:2-FA16:1	TAG
222	TAG49:2-FA17:0	TAG
223	TAG49:2-FA18:1	TAG
224	TAG49:2-FA18:2	TAG
225	TAG49:3-FA15:0	TAG

	Metabolite	Class
**226**	TAG49:3-FA16:0	TAG
**227**	TAG49:3-FA16:1	TAG
**228**	TAG49:3-FA18:2	TAG
**229**	TAG49:3-FA18:3	TAG
**230**	TAG50:0-FA14:0	TAG
**231**	TAG50:0-FA16:0	TAG
**232**	TAG50:0-FA18:0	TAG
**233**	TAG50:1-FA14:0	TAG
**234**	TAG50:1-FA16:0	TAG
**235**	TAG50:1-FA16:1	TAG
**236**	TAG50:1-FA18:0	TAG
**237**	TAG50:1-FA18:1	TAG
**238**	TAG50:2-FA14:0	TAG
**239**	TAG50:2-FA14:1	TAG
**240**	TAG50:2-FA16:0	TAG
**241**	TAG50:2-FA16:1	TAG
**242**	TAG50:2-FA18:0	TAG
**243**	TAG50:2-FA18:1	TAG
**244**	TAG50:2-FA18:2	TAG
**245**	TAG50:3-FA14:0	TAG
**246**	TAG50:3-FA14:1	TAG
**247**	TAG50:3-FA16:0	TAG
**248**	TAG50:3-FA16:1	TAG
**249**	TAG50:3-FA18:0	TAG
**250**	TAG50:3-FA18:1	TAG
**251**	TAG50:3-FA18:2	TAG
**252**	TAG50:3-FA18:3	TAG
**253**	TAG50:4-FA14:0	TAG
**254**	TAG50:4-FA14:1	TAG
**255**	TAG50:4-FA16:0	TAG
**256**	TAG50:4-FA16:1	TAG
**257**	TAG50:4-FA18:1	TAG
**258**	TAG50:4-FA18:2	TAG
**259**	TAG50:4-FA18:3	TAG
**260**	TAG50:4-FA20:4	TAG
**261**	TAG50:5-FA14:0	TAG
**262**	TAG50:5-FA16:0	TAG
**263**	TAG50:5-FA16:1	TAG
**264**	TAG50:5-FA18:1	TAG
**265**	TAG50:5-FA18:2	TAG
**266**	TAG50:5-FA18:3	TAG
**267**	TAG50:5-FA20:4	TAG
**268**	TAG51:0-FA17:0	TAG
**269**	TAG51:0-FA18:0	TAG
**270**	TAG51:1-FA15:0	TAG
**271**	TAG51:1-FA16:0	TAG
**272**	TAG51:1-FA17:0	TAG
**273**	TAG51:1-FA18:0	TAG
**274**	TAG51:1-FA18:1	TAG
**275**	TAG51:2-FA15:0	TAG
**276**	TAG51:2-FA16:0	TAG
**277**	TAG51:2-FA16:1	TAG
**278**	TAG51:2-FA17:0	TAG
**279**	TAG51:2-FA18:1	TAG
**280**	TAG51:2-FA18:2	TAG
**281**	TAG51:3-FA15:0	TAG
**282**	TAG51:3-FA16:1	TAG
**283**	TAG51:3-FA17:0	TAG
**284**	TAG51:3-FA18:2	TAG
**285**	TAG51:3-FA18:3	TAG
**286**	TAG51:4-FA15:0	TAG
**287**	TAG51:4-FA18:2	TAG
**288**	TAG51:4-FA18:3	TAG
**289**	TAG51:5-FA18:2	TAG
**290**	TAG52:0-FA16:0	TAG
**291**	TAG52:0-FA18:0	TAG
**292**	TAG52:1-FA16:0	TAG
**293**	TAG52:1-FA16:1	TAG
**294**	TAG52:1-FA18:0	TAG
**295**	TAG52:1-FA18:1	TAG
**296**	TAG52:1-FA20:0	TAG
**297**	TAG52:1-FA20:1	TAG
**298**	TAG52:2-FA14:0	TAG
**299**	TAG52:2-FA16:0	TAG
**300**	TAG52:2-FA16:1	TAG
**301**	TAG52:2-FA18:0	TAG
**302**	TAG52:2-FA18:1	TAG
**303**	TAG52:2-FA18:2	TAG
**304**	TAG52:2-FA20:0	TAG
**305**	TAG52:2-FA20:1	TAG
**306**	TAG52:2-FA20:2	TAG
**307**	TAG52:3-FA14:0	TAG
**308**	TAG52:3-FA16:0	TAG
**309**	TAG52:3-FA16:1	TAG
**310**	TAG52:3-FA18:0	TAG
**311**	TAG52:3-FA18:1	TAG
**312**	TAG52:3-FA18:2	TAG
**313**	TAG52:3-FA18:3	TAG
**314**	TAG52:3-FA20:0	TAG
**315**	TAG52:3-FA20:1	TAG
**316**	TAG52:3-FA20:2	TAG
**317**	TAG52:3-FA20:3	TAG
**318**	TAG52:3-FA22:1	TAG
**319**	TAG52:4-FA14:0	TAG
**320**	TAG52:4-FA16:0	TAG
**321**	TAG52:4-FA18:0	TAG
**322**	TAG52:4-FA18:1	TAG
**323**	TAG52:4-FA18:2	TAG
**324**	TAG52:4-FA18:3	TAG
**325**	TAG52:4-FA20:0	TAG
**326**	TAG52:4-FA20:2	TAG
**327**	TAG52:4-FA20:3	TAG
**328**	TAG52:4-FA20:4	TAG
**329**	TAG52:4-FA22:1	TAG
**330**	TAG52:4-FA22:4	TAG
**331**	TAG52:5-FA14:0	TAG
**332**	TAG52:5-FA16:0	TAG
**333**	TAG52:5-FA18:2	TAG
**334**	TAG52:5-FA18:3	TAG
**335**	TAG52:5-FA20:3	TAG
**336**	TAG52:5-FA20:4	TAG
**337**	TAG52:5-FA20:5	TAG
**338**	TAG52:5-FA22:5	TAG
**339**	TAG52:6-FA14:0	TAG
**340**	TAG52:6-FA16:0	TAG
**341**	TAG52:6-FA18:1	TAG
**342**	TAG52:6-FA18:2	TAG
**343**	TAG52:6-FA18:3	TAG
**344**	TAG52:6-FA20:4	TAG
**345**	TAG52:6-FA20:5	TAG
**346**	TAG52:6-FA22:6	TAG
**347**	TAG52:7-FA18:1	TAG
**348**	TAG52:7-FA20:5	TAG
**349**	TAG52:7-FA22:6	TAG
**350**	TAG52:8-FA16:1	TAG
**351**	TAG52:8-FA18:2	TAG
**352**	TAG53:0-FA16:0	TAG
**353**	TAG53:1-FA16:0	TAG
**354**	TAG53:1-FA17:0	TAG
**355**	TAG53:1-FA18:0	TAG
**356**	TAG53:1-FA18:1	TAG
**357**	TAG53:2-FA16:0	TAG
**358**	TAG53:2-FA17:0	TAG
**359**	TAG53:2-FA18:1	TAG
**360**	TAG53:2-FA18:2	TAG
**361**	TAG53:3-FA16:0	TAG
**362**	TAG53:3-FA17:0	TAG
**363**	TAG53:3-FA18:2	TAG
**364**	TAG53:4-FA16:0	TAG
**365**	TAG53:4-FA17:0	TAG
**366**	TAG53:4-FA18:2	TAG
**367**	TAG53:4-FA18:3	TAG
**368**	TAG53:4-FA20:4	TAG
**369**	TAG53:5-FA20:4	TAG
**370**	TAG54:0-FA18:0	TAG
**371**	TAG54:1-FA16:0	TAG
**372**	TAG54:1-FA18:0	TAG
**373**	TAG54:1-FA18:1	TAG
**374**	TAG54:1-FA20:0	TAG
**375**	TAG54:1-FA20:1	TAG
**376**	TAG54:2-FA16:0	TAG
**377**	TAG54:2-FA18:0	TAG
**378**	TAG54:2-FA18:1	TAG
**379**	TAG54:2-FA18:2	TAG
**380**	TAG54:2-FA20:0	TAG
**381**	TAG54:2-FA20:1	TAG
**382**	TAG54:2-FA20:2	TAG
**383**	TAG54:3-FA16:0	TAG
**384**	TAG54:3-FA16:1	TAG
**385**	TAG54:3-FA18:0	TAG
**386**	TAG54:3-FA18:1	TAG
**387**	TAG54:3-FA18:2	TAG
**388**	TAG54:3-FA18:3	TAG
**389**	TAG54:3-FA20:1	TAG
**390**	TAG54:3-FA20:2	TAG
**391**	TAG54:3-FA20:3	TAG
**392**	TAG54:4-FA16:0	TAG
**393**	TAG54:4-FA16:1	TAG
**394**	TAG54:4-FA18:0	TAG
**395**	TAG54:4-FA18:1	TAG
**396**	TAG54:4-FA18:2	TAG
**397**	TAG54:4-FA18:3	TAG
**398**	TAG54:4-FA20:1	TAG
**399**	TAG54:4-FA20:2	TAG
**400**	TAG54:4-FA20:3	TAG
**401**	TAG54:4-FA20:4	TAG
**402**	TAG54:4-FA22:1	TAG
**403**	TAG54:4-FA22:4	TAG
**404**	TAG54:5-FA16:0	TAG
**405**	TAG54:5-FA16:1	TAG
**406**	TAG54:5-FA18:0	TAG
**407**	TAG54:5-FA18:1	TAG
**408**	TAG54:5-FA18:2	TAG
**409**	TAG54:5-FA18:3	TAG
**410**	TAG54:5-FA20:2	TAG
**411**	TAG54:5-FA20:3	TAG
**412**	TAG54:5-FA20:4	TAG
**413**	TAG54:5-FA20:5	TAG
**414**	TAG54:5-FA22:4	TAG
**415**	TAG54:5-FA22:5	TAG
**416**	TAG54:6-FA16:0	TAG
**417**	TAG54:6-FA16:1	TAG
**418**	TAG54:6-FA18:1	TAG
**419**	TAG54:6-FA18:2	TAG
**420**	TAG54:6-FA18:3	TAG
**421**	TAG54:6-FA20:3	TAG
**422**	TAG54:6-FA20:4	TAG
**423**	TAG54:6-FA20:5	TAG
**424**	TAG54:6-FA22:5	TAG
**425**	TAG54:6-FA22:6	TAG
**426**	TAG54:7-FA16:1	TAG
**427**	TAG54:7-FA18:1	TAG
**428**	TAG54:7-FA18:2	TAG
**429**	TAG54:7-FA18:3	TAG
**430**	TAG54:7-FA20:4	TAG
**431**	TAG54:7-FA20:5	TAG
**432**	TAG54:7-FA22:5	TAG
**433**	TAG54:7-FA22:6	TAG
**434**	TAG54:8-FA18:2	TAG
**435**	TAG54:8-FA18:3	TAG
**436**	TAG54:8-FA20:4	TAG
**437**	TAG54:8-FA20:5	TAG
**438**	TAG54:8-FA22:6	TAG
**439**	TAG55:1-FA16:0	TAG
**440**	TAG55:1-FA18:1	TAG
**441**	TAG55:2-FA18:2	TAG
**442**	TAG55:3-FA18:1	TAG
**443**	TAG55:3-FA18:2	TAG
**444**	TAG55:4-FA18:1	TAG
**445**	TAG55:4-FA18:2	TAG
**446**	TAG55:5-FA18:1	TAG
**447**	TAG55:5-FA18:2	TAG
**448**	TAG55:5-FA20:4	TAG
**449**	TAG55:7-FA22:6	TAG
**450**	TAG56:1-FA16:0	TAG

	Metabolite	Class
**451**	TAG56:1-FA18:1	TAG
**452**	TAG56:2-FA16:0	TAG
**453**	TAG56:2-FA18:0	TAG
**454**	TAG56:2-FA20:0	TAG
**455**	TAG56:2-FA20:1	TAG
**456**	TAG56:3-FA16:0	TAG
**457**	TAG56:3-FA18:0	TAG
**458**	TAG56:3-FA18:1	TAG
**459**	TAG56:3-FA18:2	TAG
**460**	TAG56:3-FA20:0	TAG
**461**	TAG56:3-FA20:1	TAG
**462**	TAG56:3-FA20:2	TAG
**463**	TAG56:4-FA16:0	TAG
**464**	TAG56:4-FA18:0	TAG
**465**	TAG56:4-FA18:1	TAG
**466**	TAG56:4-FA18:2	TAG
**467**	TAG56:4-FA20:1	TAG
**468**	TAG56:4-FA20:2	TAG
**469**	TAG56:4-FA20:3	TAG
**470**	TAG56:4-FA20:4	TAG
**471**	TAG56:4-FA22:4	TAG
**472**	TAG56:5-FA16:0	TAG
**473**	TAG56:5-FA18:0	TAG
**474**	TAG56:5-FA18:1	TAG
**475**	TAG56:5-FA18:2	TAG
**476**	TAG56:5-FA20:1	TAG
**477**	TAG56:5-FA20:2	TAG
**478**	TAG56:5-FA20:3	TAG
**479**	TAG56:5-FA20:4	TAG
**480**	TAG56:5-FA22:4	TAG
**481**	TAG56:5-FA22:5	TAG
**482**	TAG56:6-FA16:0	TAG
**483**	TAG56:6-FA18:0	TAG
**484**	TAG56:6-FA18:1	TAG
**485**	TAG56:6-FA18:2	TAG
**486**	TAG56:6-FA18:3	TAG
**487**	TAG56:6-FA20:2	TAG
**488**	TAG56:6-FA20:3	TAG
**489**	TAG56:6-FA20:4	TAG
**490**	TAG56:6-FA20:5	TAG
**491**	TAG56:6-FA22:4	TAG
**492**	TAG56:6-FA22:5	TAG
**493**	TAG56:6-FA22:6	TAG
**494**	TAG56:7-FA16:0	TAG
**495**	TAG56:7-FA16:1	TAG
**496**	TAG56:7-FA18:1	TAG
**497**	TAG56:7-FA18:2	TAG
**498**	TAG56:7-FA18:3	TAG
**499**	TAG56:7-FA20:3	TAG
**500**	TAG56:7-FA20:4	TAG
**501**	TAG56:7-FA20:5	TAG
**502**	TAG56:7-FA22:6	TAG
**503**	TAG56:8-FA16:0	TAG
**504**	TAG56:8-FA16:1	TAG
**505**	TAG56:8-FA18:1	TAG
**506**	TAG56:8-FA18:2	TAG
**507**	TAG56:8-FA18:3	TAG
**508**	TAG56:8-FA20:4	TAG
**509**	TAG56:8-FA20:5	TAG
**510**	TAG56:8-FA22:5	TAG
**511**	TAG56:8-FA22:6	TAG
**512**	TAG56:9-FA18:3	TAG
**513**	TAG56:9-FA20:4	TAG
**514**	TAG56:9-FA20:5	TAG
**515**	TAG56:9-FA22:6	TAG
**516**	TAG57:2-FA18:1	TAG
**517**	TAG57:3-FA18:2	TAG
**518**	TAG58:10-FA18:2	TAG
**519**	TAG58:10-FA20:4	TAG
**520**	TAG58:10-FA22:5	TAG
**521**	TAG58:10-FA22:6	TAG
**522**	TAG58:2-FA18:1	TAG
**523**	TAG58:3-FA18:1	TAG
**524**	TAG58:5-FA18:1	TAG
**525**	TAG58:6-FA16:0	TAG
**526**	TAG58:6-FA18:0	TAG
**527**	TAG58:6-FA18:1	TAG
**528**	TAG58:6-FA20:4	TAG
**529**	TAG58:6-FA22:4	TAG
**530**	TAG58:6-FA22:5	TAG
**531**	TAG58:7-FA18:1	TAG
**532**	TAG58:7-FA18:2	TAG
**533**	TAG58:7-FA20:4	TAG
**534**	TAG58:7-FA22:6	TAG
**535**	TAG58:8-FA18:1	TAG
**536**	TAG58:8-FA18:2	TAG
**537**	TAG58:8-FA20:3	TAG
**538**	TAG58:8-FA20:4	TAG
**539**	TAG58:8-FA22:5	TAG
**540**	TAG58:8-FA22:6	TAG
**541**	TAG58:9-FA18:1	TAG
**542**	TAG58:9-FA18:2	TAG
**543**	TAG58:9-FA20:4	TAG
**544**	TAG58:9-FA22:5	TAG
**545**	TAG58:9-FA22:6	TAG
**546**	TAG60:11-FA22:6	TAG

Table S3. Plasma concentrations (μM) for representative lipid species by class. Within each lipid class, the mean, minimum, and maximum concentrations in control participants (A: Fred Hutch, B: Roswell Park) for two individual lipid metabolites are presented—the species with the lowest, or the highest, mean concentration.

A. Fred Hutch (FH)

Class	Metabolite	Mean	Min	Max
CE	CE(22:2)	0.31	0.16	0.65
CE	CE(18:2)	1761.50	991.47	2560.56
CER	CER(18:0)	0.18	0.07	0.39
CER	CER(24:0)	3.23	1.76	6.88
DAG	DAG(18:1/22:6)	0.27	0.06	1.11
DAG	DAG(18:1/18:2)	7.77	3.20	17.88
FFA	FFA(20:5)	1.13	0.48	3.33
FFA	FFA(17:0)	8.50	4.52	68.15
LPC	LPC(22:5)	0.50	0.23	1.04
LPC	LPC(16:0)	122.89	70.46	194.16
LPE	LPE(16:0)	0.91	0.57	1.73
LPE	LPE(18:0)	1.70	0.97	3.08
PC	PC(15:0/18:1)	1.08	0.54	1.99
PC	PC(16:0/18:2)	573.08	393.32	827.95
PE	PE(18:2/16:1)	0.49	0.17	1.56
PE	PE(16:0/22:6)	10.95	3.38	24.81
SM	SM(26:0)	0.65	0.46	0.98
SM	SM(22:0)	162.68	93.12	272.33
TAG	TAG52:2-FA20:0	0.17	0.07	0.32
TAG	TAG52:2-FA18:1	439.97	118.76	949.63

B. Roswell Park (RP)

Class	Metabolite	Mean	Min	Max
CE	CE(22:2)	0.25	0.16	0.58
CE	CE(18:2)	1936.11	1052.26	2922.62
CER	CER(18:0)	0.17	0.07	0.34
CER	CER(24:0)	3.70	1.27	7.07
DAG	DAG(18:1/22:6)	0.23	0.08	0.81
DAG	DAG(18:1/18:2)	8.75	2.09	31.22
FFA	FFA(24:0)	0.73	0.48	1.17
FFA	FFA(17:0)	5.74	3.29	9.64
LPC	LPC(22:5)	0.56	0.20	1.04
LPC	LPC(16:0)	138.00	80.54	206.27
LPE	LPE(16:0)	0.96	0.42	1.58
LPE	LPE(18:0)	1.72	0.71	2.80
PC	PC(15:0/18:1)	1.03	0.55	2.54
PC	PC(16:0/18:2)	628.72	338.78	939.06
PE	PE(18:2/16:1)	0.57	0.27	1.08
PE	PE(P-18:1/20:4)	9.63	2.51	24.21
SM	SM(26:0)	0.59	0.38	0.83
SM	SM(22:0)	216.67	97.73	313.71
TAG	TAG52:2-FA20:0	0.24	0.08	0.66
TAG	TAG52:2-FA18:1	538.12	182.55	2356.39

Table S4. Selected top differential metabolites by case status. 249 lipid species satisfying FDR q<0.05 in both cohorts (Figure 1B, bottom) were sorted and rank ordered by P value (FH). Beta coefficients, P values, and FDR q values derived from linear regression analyses are shown, by cohort, for the top ten metabolites.

			FH				RP			
	**Class**	**Metabolite**	**N**	**Beta**	**P**	**q**	**N**	**Beta**	**P**	**q**
**1**	TAG	TAG44:1-FA18:1	90	-1.16	5.54E-06	9.16E-04	116	-1.06	1.89E-03	3.29E-03
**2**	PC	PC(14:0/18:2)	94	-0.71	6.43E-06	9.16E-04	118	-0.64	1.28E-05	1.02E-04
**3**	TAG	TAG50:5-FA18:3	94	-0.88	7.33E-06	9.16E-04	118	-1.00	2.31E-06	4.06E-05
**4**	PC	PC(16:0/18:3)	94	-0.50	9.79E-06	9.16E-04	118	-0.60	3.13E-07	2.22E-05
**5**	CE	CE(18:4)	94	-0.79	1.23E-05	9.16E-04	118	-0.81	1.79E-05	1.27E-04
**6**	PC	PC(14:0/20:4)	92	-0.65	1.29E-05	9.16E-04	112	-0.43	2.53E-04	6.69E-04
**7**	TAG	TAG48:4-FA18:3	88	-1.04	1.38E-05	9.16E-04	117	-1.34	5.03E-07	2.22E-05
**8**	TAG	TAG50:4-FA18:3	94	-0.88	1.40E-05	9.16E-04	118	-0.95	1.36E-06	3.22E-05
**9**	PC	PC(18:2/20:3)	91	-0.66	1.64E-05	9.16E-04	117	-0.66	8.88E-06	8.08E-05
**10**	TAG	TAG46:2-FA14:0	92	-1.26	1.73E-05	9.16E-04	118	-1.36	9.82E-06	8.42E-05

Table S5. Selected top differential metabolites by case status in stage-stratified analyses. Lipid species with concentration differences in (A) early-stage (I-II) cases or (B) late-stage (III-IV) cases, relative to controls, were sorted and rank ordered by P value. Beta coefficients, P values, and FDR q values derived from linear regression analyses are shown for the top ten metabolites in each analysis.

A.

	Class	Metabolite	N	Beta	P	q
**1**	TAG	TAG56:2-FA16:0	83	-0.97	1.3E-05	6.2E-03
**2**	PC	PC(16:0/18:3)	84	-0.62	6.1E-05	6.2E-03
**3**	TAG	TAG52:4-FA18:0	84	-0.89	6.6E-05	6.2E-03
**4**	TAG	TAG54:2-FA20:0	84	-0.74	7.1E-05	6.2E-03
**5**	TAG	TAG50:3-FA18:0	83	-1.07	7.4E-05	6.2E-03
**6**	TAG	TAG52:2-FA14:0	78	-0.92	8.9E-05	6.2E-03
**7**	TAG	TAG52:8-FA16:1	76	-0.97	9.9E-05	6.2E-03
**8**	TAG	TAG54:1-FA16:0	84	-0.93	1.1E-04	6.2E-03
**9**	DAG	DAG(14:0/18:1)	76	-0.83	1.2E-04	6.2E-03
**10**	TAG	TAG50:3-FA18:3	84	-0.95	1.3E-04	6.2E-03

B.

	Class	Metabolite	N	Beta	P	FDR
**1**	CE	CE(14:0)	92	-0.81	9.7E-07	3.1E-04
**2**	TAG	TAG50:5-FA16:1	92	-1.23	1.5E-06	3.1E-04
**3**	CE	CE(14:1)	92	-0.95	1.7E-06	3.1E-04
**4**	LPC	LPC(16:0)	92	-0.37	3.1E-06	3.1E-04
**5**	TAG	TAG48:4-FA18:3	91	-1.50	3.3E-06	3.1E-04
**6**	LPC	LPC(18:0)	92	-0.45	4.3E-06	3.1E-04
**7**	TAG	TAG52:2-FA14:0	86	-1.05	4.3E-06	3.1E-04
**8**	TAG	TAG48:4-FA14:1	90	-1.16	4.5E-06	3.1E-04
**9**	TAG	TAG48:3-FA18:3	92	-1.52	6.5E-06	3.1E-04
**10**	TAG	TAG52:5-FA20:3	92	-1.06	7.2E-06	3.1E-04

Table S6. Performance characteristics for selected lipid species (Table 4) using data from FH cohort. Single lipid species were combined with CA125, age, and BMI, to classify early-stage (I-II) cases versus controls (FH cohort). Logistic regression was used to model the log odds of case status as a linear function of $\log_2$ CA125, age, and BMI, with or without a given lipid metabolite (M), after imputing missing values for each species to ½ the minimum concentration detected for that species across all participants. ROC curve analysis was conducted to estimate AUC and *spec90* conferred by the joint model (with M) versus the base model (without M: AUC=0.802, *spec90*=0.532). Species with *Δspec90*>0.05 are listed in red.

	Class	Metabolite	*spec90*	*Δspec90*	$P_{\Delta s}$	AUC	ΔAUC	$P_{\Delta A}$
**1**	DAG	DAG(16:1/18:1)	0.68	0.15	0.25	0.88	0.08	0.10
**2**	CE	CE(18:1)	0.51	-0.02	0.70	0.82	0.02	0.54
**3**	CE	CE(17:0)	0.55	0.02	0.38	0.80	0.00	0.79
**4**	DAG	DAG(20:0/20:0)	0.53	0.00		0.80	0.00	
**5**	TAG	TAG52:2-FA20:2	0.40	-0.13	0.25	0.83	0.02	0.57
**6**	TAG	TAG54:3-FA20:3	0.43	-0.11	0.31	0.84	0.03	0.46
**7**	PE	PE(P-16:0/20:3)	0.57	0.04	0.43	0.81	0.01	0.82
**8**	DAG	DAG(18:0/18:2)	0.72	0.19	0.24	0.92	0.12	0.08
**9**	TAG	TAG56:4-FA18:0	0.74	0.21	0.02	0.91	0.11	0.02
**10**	CE	CE(20:0)	0.53	0.00	1.00	0.79	-0.01	0.68

Table S7. Patient and tumor characteristics (GEO samples).

	GSE4122 (n=50)		GSE57477 (n=54)		GSE7463 (n=19)		GSE6822 (n=34)		Pooled (n=157)	
	Malignant	Benign	Malignant	Benign	Malignant	Benign	Malignant	Benign	Malignant	Benign
	32	18	37	17	9	10	28	6	106	51
Age [mean (sd)]	NA		67.1 (11.3)	62.5 (6.4)	58.4 (11.5)	63.4 (9.0)	NA			
Histology										
Serous	16	7	37	17	7	4	28	6	88	34
Non-serous	15	6	0	0	2	0	0	0	17	6
N/A	1	5	0	0	0	6	0	0	1	11
Stage										
I	1		2		1		2		6	
II	6		4		1		2		13	
III	14		26		6		20		66	
IV	0		5		1		0		6	
NA	11		0		0		4		15	

[GSE4122]

GSM94332, GSM94339, GSM94341, GSM94344, GSM94346, GSM94347, GSM94349, GSM94352, GSM94359, GSM94368, GSM94372, GSM94375, GSM94377, GSM94386, GSM94393, GSM94396, GSM94402, GSM94404, GSM94405, GSM94408, GSM94409, GSM94411, GSM94412, GSM94413, GSM94414, GSM94415, GSM94417, GSM94420, GSM94421, GSM94423, GSM94425, GSM94426, GSM94348, GSM94363, GSM94366, GSM94373, GSM94379, GSM94381, GSM94382, GSM94383, GSM94388, GSM94390, GSM94392, GSM94395, GSM94398, GSM94400, GSM94401, GSM94416, GSM94422, GSM94424

[GSE57477]

GSM1383532, GSM1383530, GSM1383529, GSM1383526, GSM1383525, GSM1383523, GSM1383522, GSM1383519, GSM1383514, GSM1383511, GSM1383507, GSM1383504, GSM1383503, GSM1383502, GSM1383501, GSM1383499, GSM1383498, GSM1383495, GSM1383494, GSM1383492, GSM1383491, GSM1383490, GSM1383488, GSM1383487, GSM1383485, GSM1383484, GSM1383482, GSM1383480, GSM1383479, GSM1383478, GSM1383476, GSM1383474, GSM1383473, GSM1383471, GSM1383469, GSM1383468, GSM1383467, GSM1383527, GSM1383524, GSM1383520, GSM1383518, GSM1383515, GSM1383513, GSM1383510, GSM1383509, GSM1383508, GSM1383506, GSM1383505, GSM1383496, GSM1383489, GSM1383481, GSM1383475, GSM1383472, GSM1383470

[GSE7463]

GSM180636, GSM180637, GSM180638, GSM180639, GSM180640, GSM180641, GSM180642, GSM180643, GSM180644, GSM180626, GSM180627, GSM180628, GSM180629, GSM180630, GSM180631, GSM180632, GSM180633, GSM180634, GSM180635

[GSE6822]

GSM157233, GSM157244, GSM157267, GSM157268, GSM157292, GSM157297, GSM157277, GSM157278, GSM157229, GSM157230, GSM157232, GSM157241, GSM157262, GSM157263, GSM157265, GSM157266, GSM157271, GSM157272, GSM157274, GSM157275, GSM157281, GSM157282, GSM157285, GSM157287, GSM157288, GSM157291, GSM157293, GSM157294, GSM157225, GSM157255, GSM157256, GSM157257, GSM157258, GSM157284

Table S8. Lipid transport-related GO gene sets selected and individual genes available for analysis. 180 GO gene sets with “lipid” or “lipoprotein” included in the set name were identified. 61 of the 180 were retained based on relationships to lipid transport. 145 of 402 unique genes captured by these 61 GO gene sets had expression profiling data available for analysis in all four GEO datasets and for subsequent meta-analysis.

**1**	GO_APOLIPOPROTEIN_A_I_BINDING
**2**	GO_APOLIPOPROTEIN_A_I_MEDIATED_SIGNALING_PATHWAY
**3**	GO_APOLIPOPROTEIN_BINDING
**4**	GO_APOLIPOPROTEIN_RECEPTOR_BINDING
**5**	GO_ATPASE_COUPLED_INTRAMEMBRANE_LIPID_TRANSPORTER_ACTIVITY
**6**	GO_ATPASE_COUPLED_LIPID_TRANSMEMBRANE_TRANSPORTER_ACTIVITY
**7**	GO_BIOACTIVE_LIPID_RECEPTOR_ACTIVITY
**8**	GO_HIGH_DENSITY_LIPOPROTEIN_PARTICLE
**9**	GO_HIGH_DENSITY_LIPOPROTEIN_PARTICLE_ASSEMBLY
**10**	GO_HIGH_DENSITY_LIPOPROTEIN_PARTICLE_BINDING
**11**	GO_HIGH_DENSITY_LIPOPROTEIN_PARTICLE_CLEARANCE
**12**	GO_HIGH_DENSITY_LIPOPROTEIN_PARTICLE_REMODELING
**13**	GO_INTERMEDIATE_DENSITY_LIPOPROTEIN_PARTICLE
**14**	GO_INTERMEMBRANE_LIPID_TRANSFER
**15**	GO_INTRACELLULAR_LIPID_TRANSPORT
**16**	GO_INTRAMEMBRANE_LIPID_TRANSPORTER_ACTIVITY
**17**	GO_LIPID_EXPORT_FROM_CELL
**18**	GO_LIPID_IMPORT_INTO_CELL
**19**	GO_LIPID_TRANSFER_ACTIVITY
**20**	GO_LIPID_TRANSLOCATION
**21**	GO_LIPID_TRANSPORTER_ACTIVITY
**22**	GO_LIPOPROTEIN_PARTICLE_RECEPTOR_ACTIVITY
**23**	GO_LIPOPROTEIN_PARTICLE_RECEPTOR_BINDING
**24**	GO_LOW_DENSITY_LIPOPROTEIN_PARTICLE
**25**	GO_LOW_DENSITY_LIPOPROTEIN_PARTICLE_BINDING
**26**	GO_LOW_DENSITY_LIPOPROTEIN_PARTICLE_CLEARANCE
**27**	GO_LOW_DENSITY_LIPOPROTEIN_PARTICLE_RECEPTOR_ACTIVITY
**28**	GO_LOW_DENSITY_LIPOPROTEIN_PARTICLE_RECEPTOR_BINDING
**29**	GO_LOW_DENSITY_LIPOPROTEIN_PARTICLE_RECEPTOR_BIOSYNTHETIC_PROCESS
**30**	GO_LOW_DENSITY_LIPOPROTEIN_PARTICLE_RECEPTOR_CATABOLIC_PROCESS
**31**	GO_LOW_DENSITY_LIPOPROTEIN_PARTICLE_REMODELING
**32**	GO_LOW_DENSITY_LIPOPROTEIN_RECEPTOR_PARTICLE_METABOLIC_PROCESS
**33**	GO_NEGATIVE_REGULATION_OF_LIPID_TRANSPORT
**34**	GO_NEGATIVE_REGULATION_OF_LIPOPROTEIN_PARTICLE_CLEARANCE
**35**	GO_NEGATIVE_REGULATION_OF_LOW_DENSITY_LIPOPROTEIN_PARTICLE_CLEARANCE
**36**	GO_PHOSPHOLIPID_TRANSFER_ACTIVITY
**37**	GO_PHOSPHOLIPID_TRANSPORT
**38**	GO_PHOSPHOLIPID_TRANSPORTER_ACTIVITY
**39**	GO_PLASMA_LIPOPROTEIN_PARTICLE_CLEARANCE
**40**	GO_POSITIVE_REGULATION_OF_LIPID_TRANSPORT
**41**	GO_POSITIVE_REGULATION_OF_LIPOPROTEIN_PARTICLE_CLEARANCE
**42**	GO_POSITIVE_REGULATION_OF_PHOSPHOLIPID_TRANSPORT
**43**	GO_REGULATION_OF_HIGH_DENSITY_LIPOPROTEIN_PARTICLE_CLEARANCE
**44**	GO_REGULATION_OF_LIPID_TRANSPORT
**45**	GO_REGULATION_OF_LIPID_TRANSPORTER_ACTIVITY
**46**	GO_REGULATION_OF_LIPOPROTEIN_PARTICLE_CLEARANCE
**47**	GO_REGULATION_OF_LOW_DENSITY_LIPOPROTEIN_PARTICLE_CLEARANCE
**48**	GO_REGULATION_OF_LOW_DENSITY_LIPOPROTEIN_PARTICLE_RECEPTOR_BINDING
**49**	GO_REGULATION_OF_LOW_DENSITY_LIPOPROTEIN_PARTICLE_RECEPTOR_CATABOLIC_PROCESS
**50**	GO_REGULATION_OF_PLASMA_LIPOPROTEIN_PARTICLE_LEVELS
**51**	GO_REGULATION_OF_VERY_LOW_DENSITY_LIPOPROTEIN_PARTICLE_CLEARANCE
**52**	GO_REGULATION_OF_VERY_LOW_DENSITY_LIPOPROTEIN_PARTICLE_REMODELING
**53**	GO_SPHERICAL_HIGH_DENSITY_LIPOPROTEIN_PARTICLE
**54**	GO_SPHINGOLIPID_TRANSFER_ACTIVITY
**55**	GO_SPHINGOLIPID_TRANSPORTER_ACTIVITY
**56**	GO_TRIGLYCERIDE_RICH_LIPOPROTEIN_PARTICLE_CLEARANCE
**57**	GO_TRIGLYCERIDE_RICH_LIPOPROTEIN_PARTICLE_REMODELING
**58**	GO_TRIGLYCERIDE_RICH_PLASMA_LIPOPROTEIN_PARTICLE
**59**	GO_VERY_LOW_DENSITY_LIPOPROTEIN_PARTICLE_ASSEMBLY
**60**	GO_VERY_LOW_DENSITY_LIPOPROTEIN_PARTICLE_CLEARANCE
**61**	GO_VERY_LOW_DENSITY_LIPOPROTEIN_PARTICLE_REMODELING

Table S8 [continued]

**1**	ABCA3	**37**	CLU	**73**	LPCAT3	**109**	PRKAA2
**2**	ABCA4	**38**	CPT1A	**74**	LPL	**110**	PRKACA
**3**	ABCC1	**39**	CRH	**75**	LRP1	**111**	PRKACB
**4**	ABCD1	**40**	CRHR1	**76**	LRP8	**112**	PRKACG
**5**	ABCD3	**41**	CRP	**77**	LRPAP1	**113**	PRKCD
**6**	ABCG1	**42**	CRY1	**78**	LSR	**114**	PTCH1
**7**	ACSL1	**43**	CSK	**79**	MAPT	**115**	PTPN11
**8**	ADIPOQ	**44**	CYP19A1	**80**	MIF	**116**	RBP4
**9**	AGT	**45**	DNAJA1	**81**	MPO	**117**	RELN
**10**	AGTR1	**46**	EPRS1	**82**	MSR1	**118**	REN
**11**	AKR1C4	**47**	FABP3	**83**	MTTP	**119**	RHOA
**12**	AKT1	**48**	FABP4	**84**	MYB	**120**	RPS6KB1
**13**	ALB	**49**	FURIN	**85**	NFKB1	**121**	S1PR1
**14**	ANXA2	**50**	GAL	**86**	NFKBIA	**122**	SCARB1
**15**	AP2B1	**51**	GM2A	**87**	NKX3-1	**123**	SCARB2
**16**	AP2M1	**52**	GPLD1	**88**	NPC1	**124**	SCARF1
**17**	AP2S1	**53**	GPR6	**89**	NPC2	**125**	SCP2
**18**	APCS	**54**	GPS2	**90**	NR1H2	**126**	SLC10A1
**19**	APOA1	**55**	HDLBP	**91**	NR1H3	**127**	SLC10A2
**20**	APOA4	**56**	HMOX1	**92**	NR1H4	**128**	SLC10A3
**21**	APOC1	**57**	HNRNPK	**93**	OSBP	**129**	SLC4A1
**22**	APOC3	**58**	HSPD1	**94**	OXT	**130**	SLCO1A2
**23**	APOD	**59**	HSPG2	**95**	P2RX4	**131**	SLCO2A1
**24**	APOE	**60**	IL1B	**96**	P2RX7	**132**	SORL1
**25**	APOH	**61**	INHBA	**97**	P4HB	**133**	SPP1
**26**	ATP5PF	**62**	ITGAV	**98**	PICALM	**134**	SREBF2
**27**	BMP1	**63**	ITGB3	**99**	PITPNA	**135**	SSTR4
**28**	BMP6	**64**	KHSRP	**100**	PITPNB	**136**	STAR
**29**	CANX	**65**	LCAT	**101**	PLA2G2A	**137**	STARD3
**30**	CD36	**66**	LDLR	**102**	PLA2G7	**138**	SYK
**31**	CDH13	**67**	LEP	**103**	PLA2R1	**139**	SYT1
**32**	CEACAM1	**68**	LIPA	**104**	PLAGL2	**140**	TAC1
**33**	CETP	**69**	LIPC	**105**	PLSCR1	**141**	THBS1
**34**	CFHR4	**70**	LPA	**106**	PON1	**142**	TMF1
**35**	CLTA	**71**	LPAR1	**107**	PPARA	**143**	TSPO
**36**	CLTC	**72**	LPAR4	**108**	PPARG	**144**	TTPA
						**145**	VLDLR

Figure S1. Lipid class assignments for 546 analyzed lipid species. ~75% are triacylglycerols (TAG). The remaining 138 species are classified as diacylglycerols (DAG), free fatty acids (FFA), cholesterol esters (CE), phosphatidylcholines (PC), phosphatidylethanolamines (PE), lysophosphatidylcholines (LPC), lysophosphatidylethanolamines (LPE), sphingomyelins (SM), and ceramides (CER).

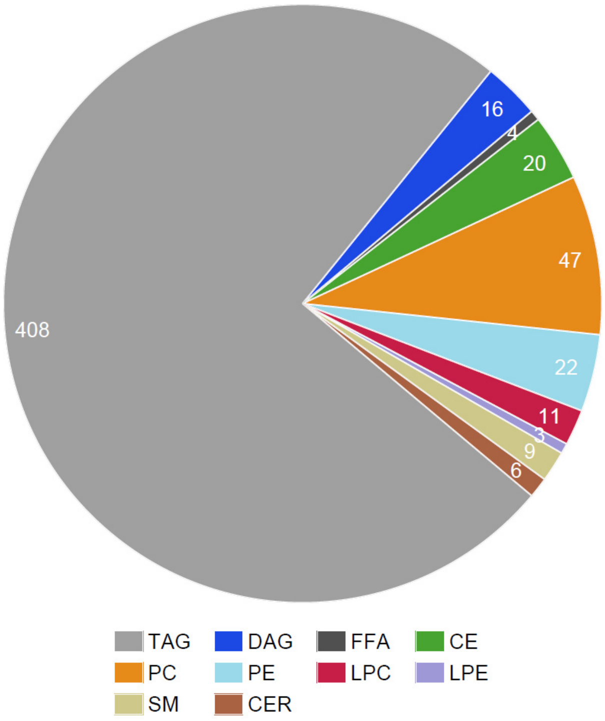

Figure S2. Distributions of coefficients of variation (CV) for 546 analyzed lipid species. Mean, minimum (Min), and maximum (Max) CV listed for each lipid class (A: Fred Hutch, B: Roswell Park).

A.

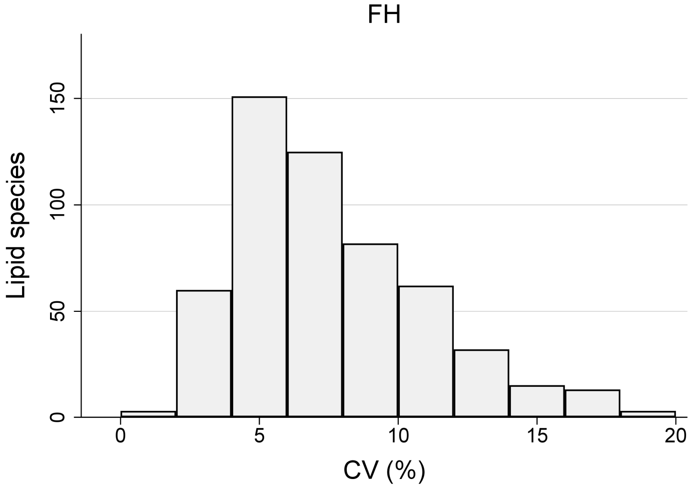

Class	N	Mean	Min	Max
CE	20	**4.0**	1.4	9.3
CER	6	**7.6**	4.4	15.4
DAG	16	**10.6**	7.7	15.3
FFA	4	**7.6**	3.1	12.0
LPC	11	**11.3**	3.8	17.8
LPE	3	**10.7**	6.9	17.5
PC	47	**10.7**	3.7	19.2
PE	22	**13.0**	7.6	17.4
SM	9	**7.9**	3.2	11.3
TAG	408	**6.8**	1.9	19.9

B.

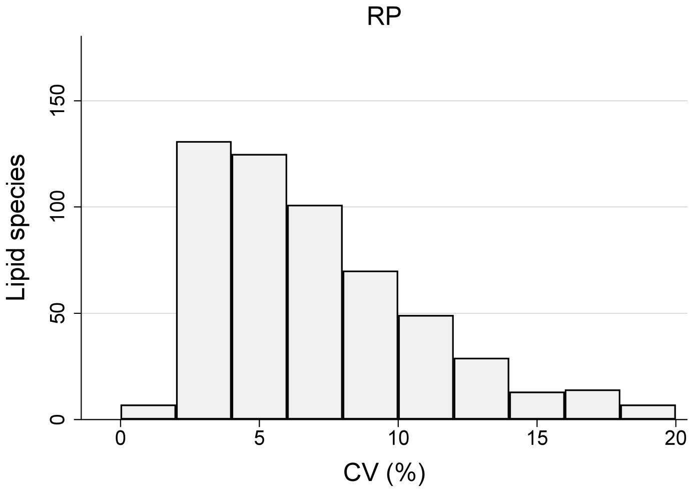

Class	N	Mean	Min	Max
CE	20	**3.8**	1.1	9.4
CER	6	**6.3**	3.8	11.1
DAG	16	**11.4**	7.7	16.0
FFA	4	**7.6**	4.5	9.5
LPC	11	**10.1**	3.7	16.7
LPE	3	**8.0**	3.7	13.1
PC	47	**8.8**	3.0	16.7
PE	22	**14.2**	7.9	19.7
SM	9	**5.4**	1.8	8.3
TAG	408	**6.4**	1.8	20.0

Figure S3. Lipid class differences in cases versus controls. Within each cohort, missing values for each species were imputed to ½ the minimum concentration detected for that species across all participants. Concentrations for all species in a given class were summed and log_2-transformed to generate class-level variables, and regressed on case status with adjustment for age and BMI. (A) All cases vs. controls, by cohort (FH: solid, RP: outlined). (B) Early-stage (I-II: "Δ") or late-stage (III-IV: "×") cases vs. controls (RP only). *Lipid classes with P<0.005 (=0.05/10) in both cohorts (A), or both subgroups (B). FFA,CER: P>0.05 (gray).

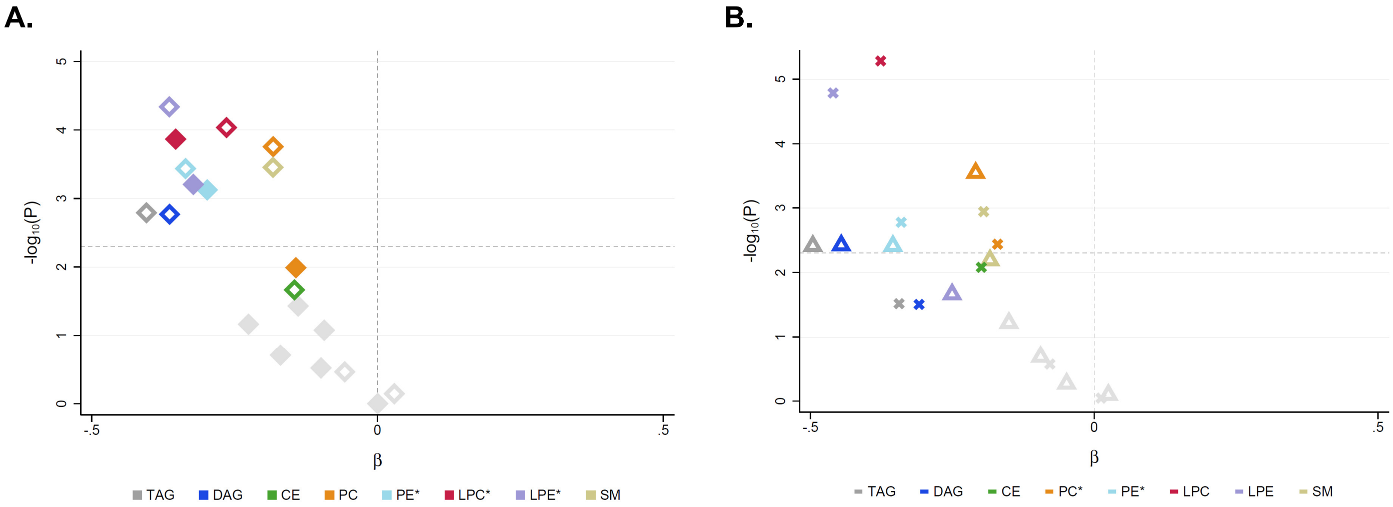

Figure S4. Plasma CA125 values (U/mL) for cases and controls. Distributions for both early-stage (blue) and late-stage (red) cases, as indicated.

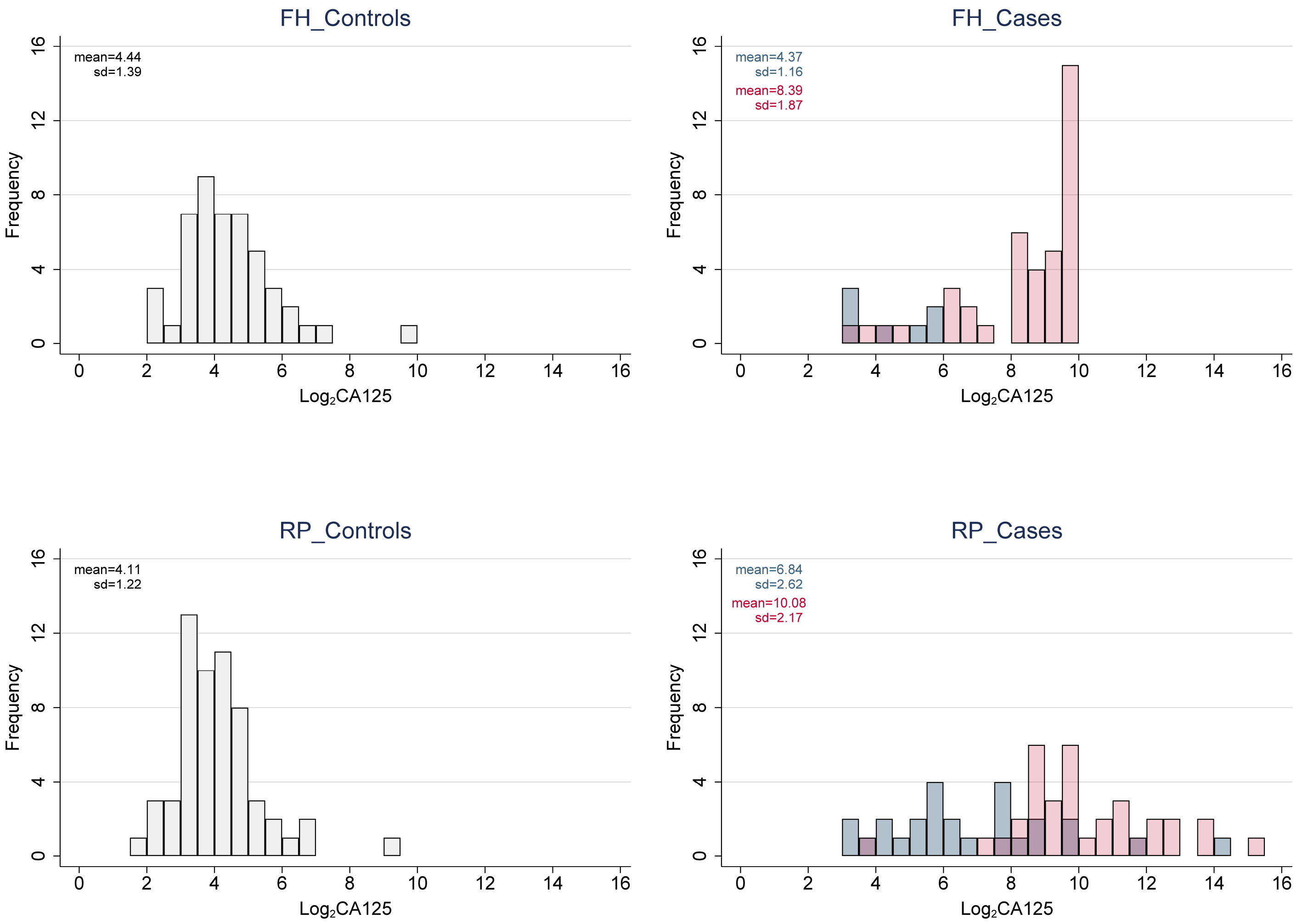

**NOTE: N=14 CA125 values >1000 U/mL (FH) could not be further resolved due to limited remaining plasma and were recorded as ≥1000 U/mL.*

Figure S5. ROC curves and performance metrics for base models comprised of CA125, age, and BMI. Models were built using data from all cases and controls in the Fred Hutch (A) or Roswell Park (B) patient cohorts. Performance was assessed across all patients in a given cohort (left), or in subgroups (right) comprised of controls and early-stage cases (blue), or controls and late-stage cases (red).

A. Fred Hutch (FH)

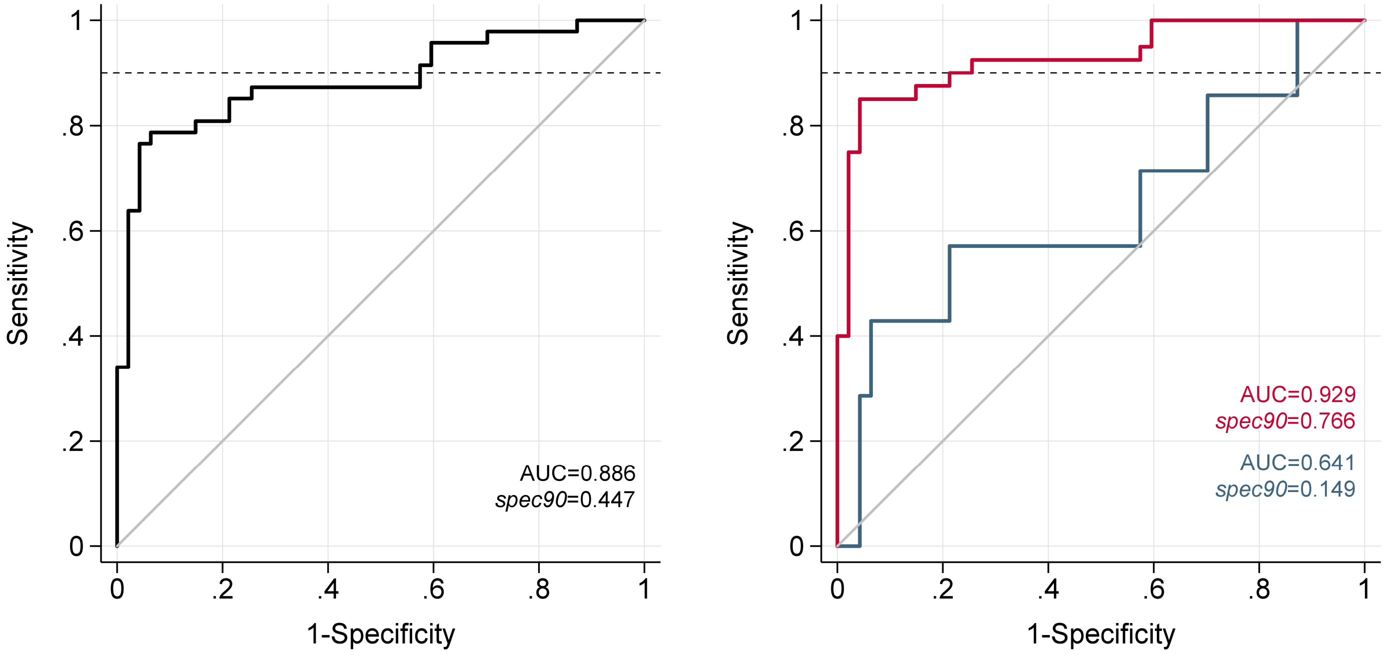

B. Roswell Park (RP)

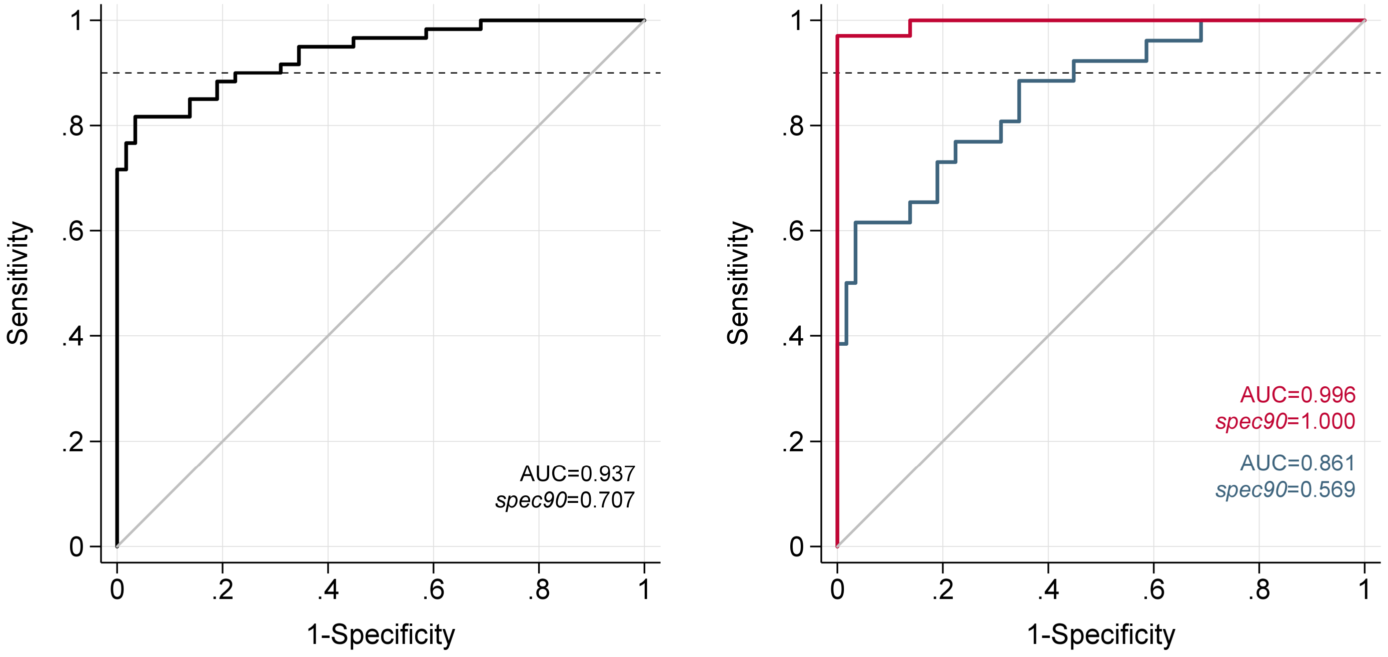

Figure S6. Spearman correlation analysis of lipid concentrations in paired ascites and plasma specimens isolated from 15 patients with late-stage OC. Correlation coefficients (rho) plotted for >500 individual lipid species analyzed (gray: |rho|<0.2, blue: |rho|≥0.2). 16 metabolties with $P_{rho}<0.05$ are marked (*).

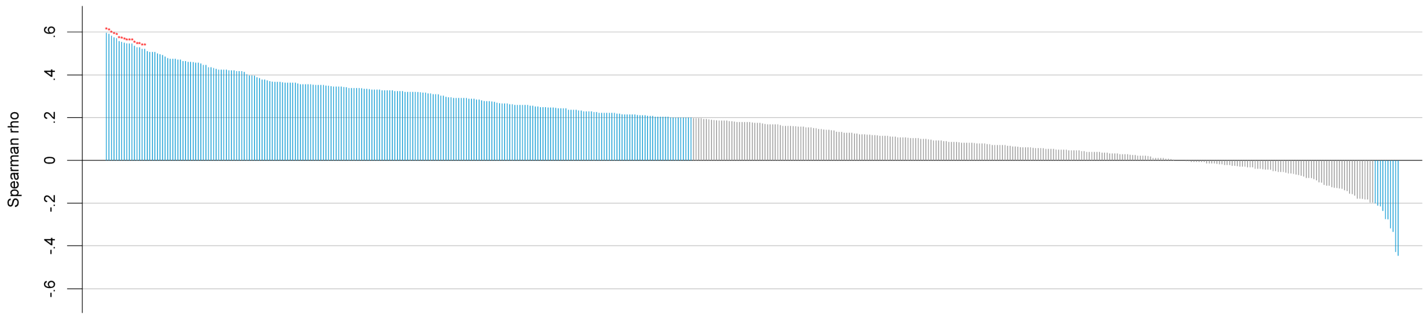

Figure S7. Schematic summary. Relative differences in plasma lipid metabolite levels and tissue gene expression levels in women with malignant, versus benign, adnexal mass.

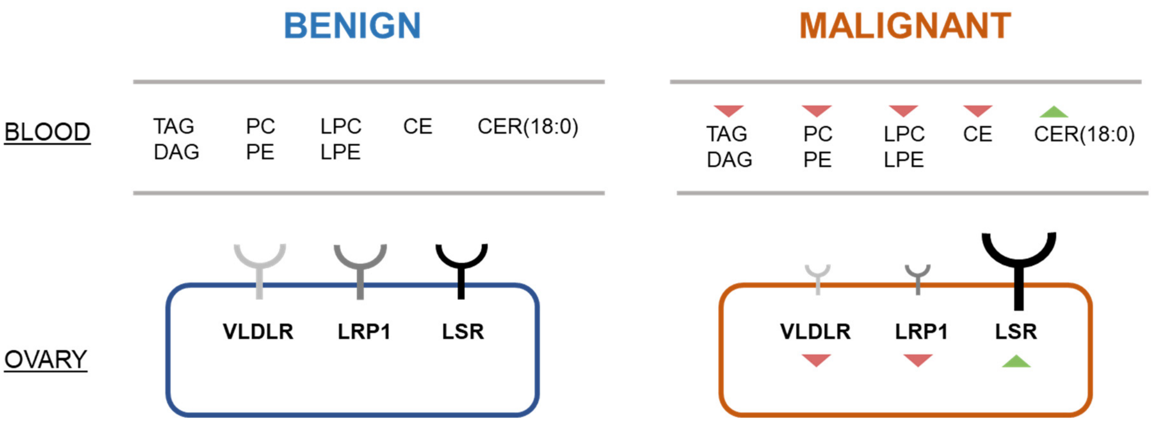

Supplement: Supplementary file 1 — Supplementary Information. [file 41598_2021_97433_MOESM1_ESM.pdf]
